# Cellular sensor DAP5 decodes *Betacoronaviral* NSP5 to drive virus-induced senescence

**DOI:** 10.3389/fimmu.2026.1768183

**Published:** 2026-03-17

**Authors:** Yao Lu, Jun Xiao, Jiale Wang, Zihan Meng, Guangyue Fan, Jian Zheng, Minghang Yu, Xi Wang, Long Li

**Affiliations:** 1 Tianjin Institute of Immunology, Key Laboratory of Immune Microenvironment and Disease (Ministry of Education), State Key Laboratory of Experimental Hematology, Department of Immunology, Tianjin Medical University, Tianjin, China; 2 State Key Laboratory of Experimental Hematology, National Clinical Research Center for Blood Diseases, Tianjin Institute of Immunology, Haihe Laboratory of Cell Ecosystem, Institute of Hematology and Blood Diseases Hospital, Chinese Academy of Medical Sciences and Peking Union Medical College, Tianjin, China; 3 National Key Laboratory of Intelligent Tracking and Forecasting for Infectious Diseases, Beijing Institute of Infectious Diseases, Beijing Key Laboratory of Viral Infectious Diseases, Beijing Ditan Hospital, Capital Medical University, Beijing, China

**Keywords:** DAP5, NSP5, TRIM7, viral replication, virus-induced senescence

## Abstract

**Background:**

Viral infection induces host cells to enter a state of “virus-induced senescence (VIS)”, which provides a stable cellular environment for viral replication. However, it is unclear about the molecular mechanism of this process. Here, we identified cellular protein DAP5 and its N-terminal fragment DAP5_1–451_ as sensors to viral infection.

**Methods:**

Upon SARS-CoV-2 infection, cellular apoptosis and senescence levels were assessed. This led to the identification of DAP5 as a pivotal proteolytic substrate that links viral protease activity to host cell fate determination. The specific cleavage site on DAP5 targeted by the non-structural protein 5 (NSP5) encoded by SARS-CoV-2 was mapped using Western Blot and Fluorescence Resonance Energy Transfer (FRET) analysis. The functional role of the resulting N-terminal fragment DAP5_1–451_ was then characterized through a series of molecular biology experiments, including ChIP-seq, dual-luciferase reporter assays, and co-immunoprecipitation (Co-IP). Furthermore, ubiquitination assays and protein stability analyses were conducted to delineate the degradation pathway responsible for clearing this N-terminal fragment DAP5_1-451_.

**Results:**

Viral infection-activated caspase 3 cleaves DAP5, which contributed to positive feedback loops, reinforcing apoptotic process. NSP5 interrupted the apoptotic process by NSP5-specific cleavage of DAP5 that led to the production of the N-terminal fragment DAP5_1-451_, which initiated the cellular senescence program, achieving an “apoptosis→senescence” fate transformation and thereby promoting viral replication of SARS-CoV-2. Mechanistically, DAP5_1–451_ interacted with the transcription factor p53 to enter the nucleus and bind to *CDKN1A* locus to increase its expression, thereby triggering cell cycle arrest. Additionally, DAP5_1–451_ activated the NF-κB signaling pathway and promoted the production of senescence-associated secretory phenotype (SASP) factors. E3 ubiquitin ligase TRIM7 encoded by host cells degraded the N-terminal fragment DAP5_1–451_ by Glutamine C-degron-mediated ubiquitination and protein degradation, and restricted viral replication.

**Conclusions:**

Our findings clarify the mechanism of SARS-CoV-2 induced VIS and establish a model of host cells inhibiting VIS through protein degradation and limiting viral replication, which provides a basis for subsequent immunological studies of emergent pathogenic microbial infection.

## 1 Introduction

COVID-19, caused by severe acute respiratory syndrome coronavirus 2 (SARS-CoV-2), has been a significant threat to human health. SARS-CoV-2 encodes 29 proteins, including 16 nonstructural proteins (NSPs) that play crucial roles in immune evasion as well as in viral RNA replication and transcription (1, 2). Among these NSPs, NSP5 (also known as 3-Chymotrypsin-like protease [3CLpro]) processes viral polyproteins at 11 cleavage sites to generate NSP4-NSP16, which makes it the main protease (Mpro) for the virus (3). NSP5/3CLpro by nature is an endopeptidase that recognizes the Leu-Gln motif and cleaves after the Gln residue to generate Gln C-termini for its products. Beyond its essential role in processing of viral polyproteins, NSP5 targets host proteins as well, including TAB1, NLRP12, RIG-I, NEMO (4–8), to interfere with host antiviral mechanisms.

Cellular stress responses, such as oxidative stress and DNA damage, are induced upon viral infection, which leads to apoptosis and is a critical antiviral mechanism that suppresses viral replication (9, 10). However, viruses have evolved a variety of counter-mechanisms to evade apoptosis (11, 12) to sustain the viability of host cells for promoting viral replication and persistence, including virus-induced senescence (VIS). In contrast to apoptosis, VIS does not lead to cell death but instead creates a metabolically active yet non-dividing cellular state that offers a stable environment conducive to viral replication and persistence. VIS has been identified in cells infected with SARS-CoV-2 (13–15). VIS is characterized by a high resistance to apoptosis, irreversible cell cycle arrest, senescence-associated secretory phenotype (SASP) (16) and increased β-galactosidase activity. Mechanistically, cellular stresses caused by viral infection induce elevated expression of p53 (encoded by *TP53*), whose transcriptional targets include cyclin-dependent kinase (CDK) inhibitors p21^CIP1^ (p21, encoded by *CDKN1A*) (17). p21, together with p16^INK4A^ (p16, encoded by *CDKN2A*), leads to resistance to apoptosis and cell cycle arrest (18). SASP is characterized by the production of pro-inflammatory cytokines, extracellular matrix regulators and pro-coagulant factors, and is partially controlled by NF-κB (14, 16, 19). Therefore, NF-κB and the above-mentioned cell cycle regulators collectively form the core of the genetic regulatory network that drives VIS.

The other side of the viral-host interactions is the cellular coordination for pro-apoptotic response, with DAP5 (also called p97 or NAT1) being a vital molecule in this process. DAP5 is a member of the eIF4G protein family that mediates the initiation of mRNA translation, especially the IRES-driven cap-independent translation (20). Under apoptotic conditions, it is cleaved at _789_DETD_792_ by caspase 3 to generate a DAP5/p86 truncated protein to enhance the IRES-mediated translation of apoptosis associated proteins, such as Apaf-1 and XIAP, to fine tune the balance between cell death and viability (21–23). Some viruses have evolved mechanisms to take advantage of DAP5. For example, coxsackievirus B3 (CVB3) 2A protease cleaves DAP5 at G434, generating two fragments that cooperate to promote viral progeny release by enhanced apoptosis resulting from altering translation of IRES-containing genes (24). We have identified several putative NSP5 recognition sites within DAP5 (**Supplementary Figure 1**), raising the possibility that NSP5-specific cleavage of DAP5 contributes to survival and replication of SARS-CoV-2 in host cells.

Our previous work has demonstrated that cellular E3 ligase TRIM7 recognizes its substrates through a C-terminal Gln-containing motif to catalyze ubiquitination and promote degradation (25). Since the C-termini of proteolytic products of NSP5 are Gln, we speculated that the fragments of DAP5 generated by endopeptidase activity of NSP5 with Gln being their C-termini may be targeted for ubiquitination for degradation by TRIM7. We further speculated that the TRIM7 induced degradation of cleaved DAP5 contributed to cellular antiviral response upon SARS-CoV-2 infection.

To model the initial phase of natural infection, we employed a low multiplicity of infection (MOI), early-infection paradigm (within 48 hpi). Under these conditions, we demonstrated that SARS-CoV-2-induced VIS, and found that cleaved fragment of DAP5 by NSP5 was an important molecular driver for VIS. TRIM7 catalyzed ubiquitination of cleaved DAP5 and inhibited the proliferation of SARS-CoV-2. The interactions among NSP5, DAP5 and TRIM7 identified in this report may be a target of future search for inhibitors of *Betacoronaviruses*.

## 2 Methods

### 2.1 Cell culture and plasmid transfection

HEK293T cells were cultured in DMEM (Gibco) supplemented with 10% fetal bovine serum (FBS, ExCell) and 1% penicillin-streptomycin (Beyotime). A549 cells were maintained in RPMI-1640 medium (Gibco) supplemented with 10% FBS, 1% penicillin-streptomycin, and 1% non-essential amino acids. Caco-2 cells were cultured in DMEM containing 20% FBS and 1% penicillin-streptomycin under the same conditions. All cell lines were incubated at 37 °C with 5% CO_2_. Mouse embryonic fibroblasts (MEFs) were isolated and cultured as follows: embryos at embryonic day 13.5 (E13.5) were collected, and the head and internal organs were removed. The remaining tissues were minced and digested with 0.05% trypsin (Beyotime), with gentle pipetting every 5 minutes to enhance digestion. After 15 minutes, digestion was terminated by adding a complete medium. Four hours after seeding, cell attachment was observed under a microscope, and the medium was replaced with DMEM containing 10% FBS and 1% penicillin-streptomycin. Cells were cultured until they reached 90% confluence and were then cryopreserved.

For transfection, cells were pretreated with DMEM containing 1% FBS for 3 hours. A transfection mixture containing opti-MEM (Gibco), plasmid DNA, and Lipo8000 (Beyotime) was prepared and incubated with cells for 6 hours. Afterward, the medium was replaced with a fresh, complete medium. Transfection efficiency was assessed using fluorescence microscopy, Western blotting, and flow cytometry. Inhibitors and activators were listed in **Supplementary Table 1**.

### 2.2 Flow cytometry

For the Fluorescence Resonance Energy Transfer (FRET) and protein stability assay, cells were harvested and thoroughly washed with PBS. Dead cells were identified using NIR Zombie dye staining. Following the initial labeling, cells were rewashed with flow cytometry-grade PBS to remove excess dye. Fluorescence signals were subsequently analyzed using a BD FACS Canto II flow cytometer.

For the apoptosis assay, cells were double-stained with Annexin V and PI using the apoptosis detection kit (Pricella, P-CA-203) according to the manufacturer’s instructions. Immediately following staining, fluorescence signals were analyzed using flow cytometry.

For cell cycle assay, cells were labeled with EdU for 1 hour and then harvested. EdU staining was performed using the Click reaction according to the manufacturer’s protocol (Beyotime, BeyoClick™ EdU Cell Proliferation Kit with Alexa Fluor 647). After EdU staining, PI staining solution was added and incubated for 15 minutes. Fluorescence signals were subsequently analyzed by flow cytometry. FlowJo V10 was used for data analysis.

### 2.3 Western blot

Cells were lysed on ice for 30 minutes in RIPA lysis buffer (Millipore) supplemented with protease inhibitors (Roche) and PMSF (Solarbio), with vortexing every 10 minutes to ensure complete lysis. The lysate was centrifuged at 12,000 g for 15 minutes at 4 °C, and the supernatant was mixed with 5× SDS-PAGE loading buffer (GenStar). The mixture was heated at 95 °C for 10 minutes to prepare protein samples.

Proteins were separated by SDS-PAGE and transferred to the PVDF membrane (Millipore) at 300 mA for 110 minutes. The membrane was blocked with TBST containing 5% BSA (Solarbio) for 2 hours at room temperature. Afterward, it was incubated overnight at 4 °C with the primary antibody (diluted according to the manufacturer’s instructions). After washing the membrane three times with TBST, it was incubated with HRP-conjugated secondary antibody at room temperature for 1 hour. After additional washes, chemiluminescent signals were detected using the StarSignal Chemiluminescent Assay Kit (GenStar) and imaged with the GelView9000 Lite imaging system (BLT). Quantification of band intensity was performed with ImageJ. The antibodies used are listed in **Supplementary Table 2**.

### 2.4 SA-β-gal staining

The β-galactosidase staining solution was prepared with 5 mM potassium ferrocyanide, 5 mM potassium ferricyanide, 1 mg/mL X-gal, 150 mM NaCl, and 2 mM MgCl_2_. Cells were fixed with a fixation solution containing 2% paraformaldehyde and 0.2% glutaraldehyde in PBS for 15 minutes. After fixation, cells were washed three times with PBS to remove residual fixative. The β-galactosidase staining solution was added, and the cells were incubated at 37 °C for 8–12 hours in the dark to avoid light interference during the reaction. Following the staining reaction, the excess solution was gently removed with PBS. The staining results were observed under a microscope, with positive β-galactosidase activity appearing as blue regions.

### 2.5 RNA extraction and RT-PCR

Total RNA was extracted from cells using Trizol (GenStar) according to the manufacturer’s protocol. Reverse transcription was performed according to the instructions of the TransScript cDNA Synthesis Kit (Transgene). After reverse transcription, RT-PCR was conducted using the LightCycler^®^ 96 system (Roche). Relative gene expression levels were calculated by the ΔΔCt method, normalized to a housekeeping gene. All primers for RT-PCR used in this study are listed in **Supplementary Table 3**.

### 2.6 Co-IP

The NP-40 lysis buffer was prepared with 50 mM Tris-HCl, 150 mM NaCl, 1% NP-40, protease inhibitors (Roche), and PMSF. Cells were lysed on ice for 30 minutes in NP-40 lysis buffer, vortexing every 10 minutes to ensure complete lysis. The lysate was centrifuged at 12,000 g for 15 minutes at 4 °C to remove debris, and the supernatant was collected.

For immunoprecipitation, the supernatant was incubated with Anti-Flag affinity gel, Anti-HA affinity gel, or Anti-His magnetic beads (Beyotime) at 4 °C for 4 hours to allow efficient binding of the target protein. The gel/beads were washed three times with TBS to remove unbound proteins. To elute the bound proteins, competitive elution was performed to dissociate proteins from the gel/beads, and the supernatant was mixed with 5× SDS-PAGE loading buffer. Alternatively, 5× SDS-PAGE loading buffer was directly added to the gel/bead-protein mixture, followed by heating at 95 °C for 5 minutes to release the proteins. Protein samples were then prepared for Western blot analysis to evaluate protein-protein interactions.

### 2.7 Construction of shRNA and sgRNA expression plasmids

All shRNAs used in this study are listed in **Supplementary Table 4**. The primers were prepared at a concentration of 10 μM. For annealing, a reaction mixture was prepared as follows: 4.25 μL of Oligo-F, 4.25 μL of Oligo-R, 1 μL of 10× T4 Ligation Buffer, and 0.5 μL of T4 Polynucleotide Kinase (PNK, Thermo Scientific). The reaction was incubated at 37 °C for 30 minutes, followed by heating at 95 °C for 5 minutes. The temperature was then gradually reduced to 25 °C at a rate of 5 °C per minute to complete the annealing process. The annealed product was diluted 1:80 for subsequent use. The pLV-shRNA plasmid was digested with BamHI and EcoRI restriction enzymes (Thermo Scientific). The digested plasmid was separated by agarose gel electrophoresis, and the approximately 7,000 bp band was excised and purified using an Agarose Gel Extraction Kit (TransGen). A ligation reaction was prepared in a total volume of 10 μL, containing 50 ng of the digested plasmid, 1 μL of the diluted annealed primers, 1 μL of 10× T4 Ligation Buffer, 0.5 μL of T4 Ligase, and sterile nuclease-free water. The reaction was incubated at room temperature for 4 hours, followed by transformation into competent cells. Single colonies were picked for sequencing verification. Plasmid DNA was extracted from the bacterial culture using a Plasmid Extraction Kit (TIANGEN) following the manufacturer’s instructions. The shRNA plasmids targeting *DAP5*, *CDKN1A* and *TP53* were generated following the same procedure described above.

For targeting the NSP5 cleavage site within DAP5, the sgRNA expression plasmids were constructed using the PX458 vector. The sgRNA target sequences are listed in **Supplementary Table 5**. The plasmid was constructed using standard molecular cloning techniques.

### 2.8 Construction of HEK293T cell line with endogenous DAP5 mutations

HEK293T cells were transfected with a mixture of opti-MEM, plasmid, and Lipo8000 transfection reagent, and incubated for 24 hours. GFP-positive cells were sorted using a flow cytometer and transferred to a 96-well plate, with one GFP-positive cell per well, for further culture. After approximately 15 days, RNA was extracted from the single-cell clones, and cDNA was synthesized via reverse transcription. PCR amplification was performed using the following primers:

Forward: 5’- AGGTAGCGGAATTGGTACTGG -3’.
Reverse: 5’- GGCCTCAGGCTAATCTCATCTGC-3’.

The PCR products were sequenced to identify and screen for single-cell clones with mutations at the NSP5 cleavage site. Ultimately, two mutant cell lines were obtained: DAP5-mut1, carrying an in-frame deletion at residue L450, and DAP5-mut2, harboring an in-frame deletion spanning residues 451-453 (_451_QGQ_453_). The sequencing results are shown in **Supplementary Figure 5A**.

### 2.9 SARS-CoV-2 infection-related experiments

The SARS-CoV-2 GFP/ΔN trVLP was generously provided by Dr. Qiang Ding at Tsinghua University. Cells were infected with SARS-CoV-2 or SARS-CoV-2 GFP/ΔN trVLP at an MOI of 0.1 or 1, respectively. All experiments involving virus infections were performed in the biosafety level 3 facility of Beijing Ditan Hospital, following all regulations.

### 2.10 Immunofluorescence staining

Glass slides pre-coated with poly-L-lysine (Solarbio) were placed in a 12-well plate, and cells were cultured until they reached 70–80% confluence. The slides were removed for further processing after performing plasmid transfection or viral infection experiments.

Cells were fixed with 4% paraformaldehyde at room temperature for 10 minutes and then washed with PBS to remove residual fixative. The cells were permeabilized with 0.25% Triton X-100 for 10 minutes and washed with PBS. To block nonspecific binding, 1% BSA was added, and the cells were incubated for 1 hour. Next, primary antibodies were applied, and the slides were incubated overnight at 4 °C. After washing away unbound primary antibodies with PBST (PBS containing 0.05% Tween 20), fluorescently labeled secondary antibodies matching the primary antibody’s host species were added. The slides were incubated at room temperature for 1 hour and washed with PBST to remove unbound secondary antibodies. DAPI (Solarbio) was used to stain cell nuclei by incubating the slides for 5 minutes, followed by washing with PBST to remove excess dye. Finally, the slides were mounted with an anti-fade mounting medium (Solarbio) and observed under an Olympus FV1000 confocal microscope.

### 2.11 ChIP-seq and ChIP-qPCR

HEK293T cells were cultured to 70–80% confluence and transfected with the Flag-DAP5_1–451_ plasmid. A control group was transfected with the DAP5_1–451_ plasmid. The DAP5_1–451_ overexpression plasmids (both FLAG-tagged and untagged versions) were generated *de novo* in this study. Cross-linking and immunoprecipitation using anti-Flag affinity gel were performed according to the manufacturer’s protocols of the ChIP Assay Kit (Beyotime). ChIP sequencing was performed on the Illumina NovaSeq 6000 platform using paired-end reads (150 bp). The raw sequencing data generated in this study have been deposited in the NCBI Sequence Read Archive (SRA) database under the BioProject accession number PRJNA1418836.

For ChIP-seq analysis, quality-controlled FASTQ files were aligned to the human reference genome (hg19). After removing duplicate reads, significant protein-binding regions in the ChIP-seq data were identified using MACS2. The BAM files were converted into BigWig format for visualization using the bamCoverage tool from deepTools. Peak annotation was performed using Homer, aligning the peaks to the human reference genome. BigWig files were uploaded to the UCSC Genome Browser for visualization. Public datasets GSM3444907, GSM5903250, and GSM5903247 were used to analyze genome epigenetic modifications.

For ChIP-qPCR, the effectiveness of ChIP-enriched genomic DNA fragments was validated by real-time quantitative PCR. Primers were designed to target the *CDKN1A* promoter region. Data were normalized to the value of the group HEK293T cells transfected with DAP5_1–451_ plasmids.

### 2.12 Dual-luciferase reporter gene assay

For DAP5_1–451_ functional analysis, the sequences of Peak1 and Peak2 relative to the *CDKN1A* promoter were inserted into the Firefly luciferase plasmid to construct Firefly luciferase reporter plasmids (The coordinates of Peak1 and Peak2 are provided in **Supplementary Table 6**). The constructed Firefly luciferase reporter plasmid and Renilla luciferase reporter plasmid were co-transfected into HEK293T cells with DAP5_1-451_. Cells were collected 24–48 hours post-transfection.

For NF-κB transcription factor activity detection, a 3×NF-κB response element sequence was inserted into the Firefly luciferase plasmid upstream of the SV40 promoter to construct the Firefly luciferase reporter plasmid. The Firefly luciferase reporter plasmid and Renilla luciferase reporter plasmid were transfected into HEK293T cells, followed by either plasmid transfection or SARS-CoV-2 infection. Cells were collected 24 hours post-transfection.

Firefly and Renilla luciferase activities were measured using a luciferase assay kit (Beyotime), and the data were recorded by GBOXiChemiXT (BioTek). The normalized results were obtained by calculating the ratio of Firefly luciferase activity to Renilla luciferase activity.

### 2.13 RNA-seq

We collected the public gene expression dataset GSE157103 to investigate the relationship between TRIM7 gene expression and the severity of COVID-19. The dataset was downloaded from the GEO database (www.ncbi.nlm.nih.gov/geo/). The raw “counts” values from the GSE157103 dataset were already transformed into Transcripts Per Million (TPM) values in the GEO database. We used the “ggboxplot” function of the “ggpubr” R package to demonstrate TRIM7 gene expression value in severe (ICU: yes) and non-severe (ICU: no) COVID-19 patients. The Wilcoxon rank-sum test was used to compare the two groups, and a two-sided P < 0.05 was considered statistically significant.

### 2.14 Enzyme-linked immunosorbent assay

Levels of secreted IL-8 in cell culture supernatants were measured using LiankeBio ELISA kits (EK108) strictly following the manufacturer’s instructions. Concentrations were determined based on a standard curve.

### 2.15 Mice

The hACE2 transgenic C57BL/6 mice were purchased from Cyagen and used to extract MEF cells (ACE2-MEF in the article). They were bred and maintained under SPF conditions at the Experimental Animal Center of Tianjin Medical University. All procedures conformed to the university’s animal care and use guidelines.

### 2.16 Statistical analysis

This study assessed the significance of differences between the two groups using an unpaired two-tailed Student’s *t*-test. ANOVA was performed for comparisons involving three or more groups, followed by Bonferroni’s *post hoc* test for intergroup differences. Statistical significance was set at *p* < 0.05. The significance levels were set as *p* < 0.05 (*), *p* < 0.01 (**), *p* < 0.001 (***), and *p* < 0.0001 (****). All data presented are representative results from multiple independent experiments or combined data from various replicates.

## 3 Results

### 3.1 SARS-CoV-2 diverts cell fate from apoptosis to senescence to enhance viral replication

**Figure 1:**
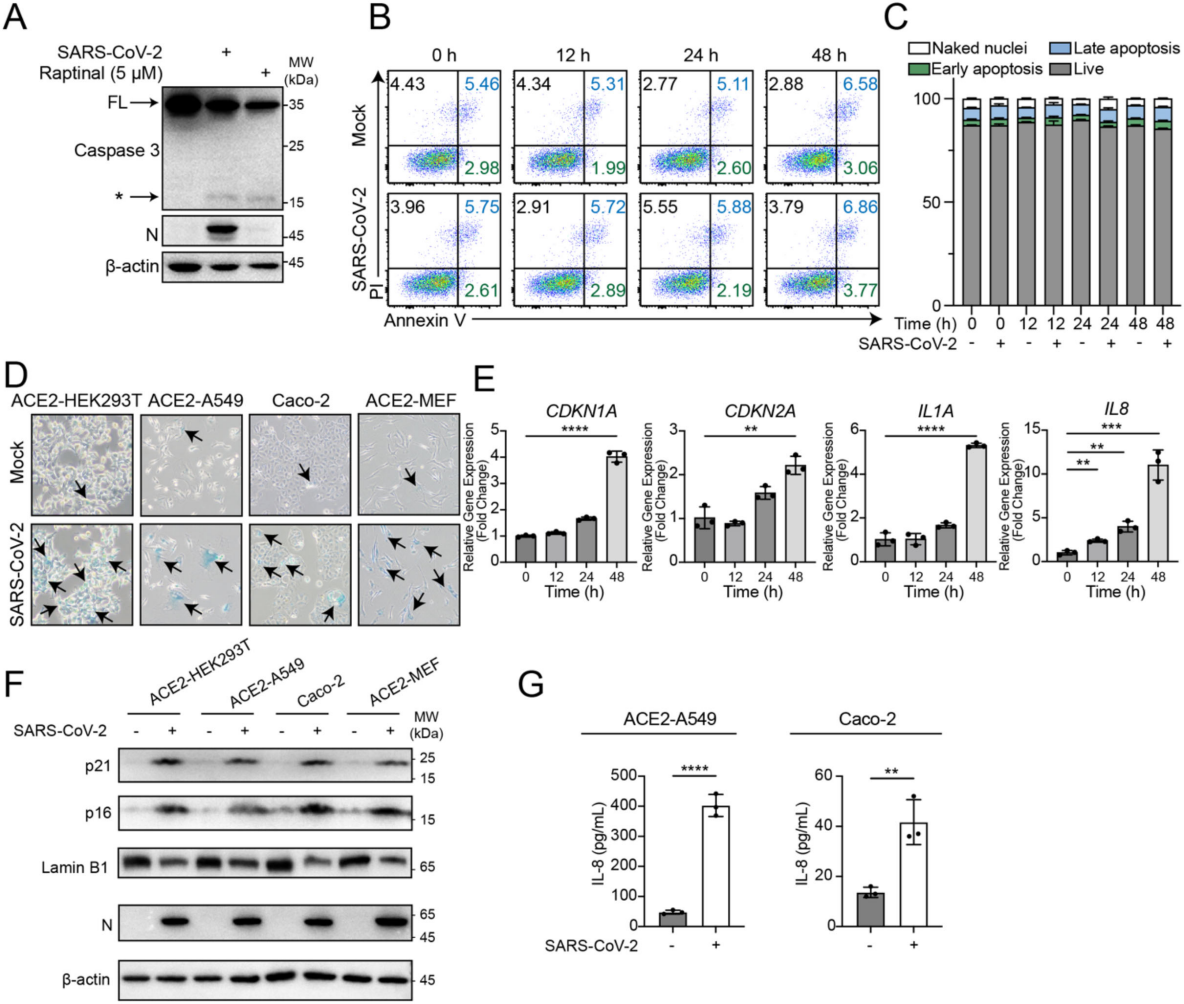
Analysis of apoptosis and senescence in ACE2-HEK293T cells following SARS-CoV-2 infection. **(A)** Western blot analysis of caspase 3 cleavage in ACE2-HEK293T cells infected with SARS-CoV-2 (MOI = 0.1) for 24 hours. Caspase 3 activator raptinal (5 μM) was used as a positive control, with treatment for 6 hours. Viral infection was confirmed by Nucleocapsid (N) detection. The black asterisk indicates cleaved caspase 3. **(B, C)** Representative flow cytometry analysis **(B)** and statistical analysis **(C)** of apoptosis using Annexin V and PI staining in ACE2-HEK293T cells infected with SARS-CoV-2 (MOI = 0.1) at 0, 12, 24, and 48 hours post-infection. **(D)** The β-galactosidase activity of cells at 48 hours post-infection with SARS-CoV-2 (MOI = 0.1). Senescent cells are stained blue, as indicated by arrows. **(E)** The expression of senescence-associated genes *CDKN1A*, *CDKN2A*, *IL1A*, and *IL8* in ACE2-HEK293T cells infected with SARS-CoV-2 (MOI = 0.1). **(F)** Western blot analysis of p21, p16, Lamin B1 and Nucleocapsid (N) expression in ACE2-HEK293T, ACE2-A549, Caco-2, and ACE2-MEF cells, either mock-treated or infected with SARS-CoV-2 at an MOI of 0.1 for 48 hours. **(G)** ELISA analysis of secreted IL-8 in cell culture supernatants of ACE2-A549 and Caco-2 cells at 48 hours after mock treatment or infection with SARS-CoV-2 (MOI = 0.1). Each experiment was independently repeated three times with similar results, and the representative images were shown. Mean ± SEM. **P < 0.01; ***P < 0.001; ****P < 0.0001.

To model the early phase of natural infection, cells were infected with SARS-CoV-2 at a low multiplicity of infection (MOI) and analyzed within 48 hours post-infection. We dissected apoptosis in cells infected with SARS-CoV-2. Cleaved caspase 3, the major apoptotic effector in the infected cells, was detected, indicating a successful initiation of apoptotic pathway by SARS-CoV-2 (**Figure 1A**). However, the infection did not induce phenotypic apoptosis in ACE2-HEK293T, ACE2-A549, Caco-2, or ACE2-MEF cells when assayed with the surrogate apoptotic biomarkers Annexin V and propidium iodide (PI) (**Figures 1B, C**; **Supplementary Figure 2**). Instead, the infected cells exhibited strong signals for β-galactosidase activity and enhanced expression of senescence markers such as *CDKN1A*, *CDKN2A*, *IL1A*, and *IL8*, along with reduced protein levels of Lamin B1 (**Figures 1D–G**), indicating that SARS-CoV-2 induced VIS. Given that senescent cells exhibit resistance to apoptosis (13, 26, 27), we speculated that under the low-MOI, early-infection paradigm used here, SARS-CoV-2 shifted the fate of infected cells from apoptosis to senescence for its survival advantage.

Although cellular senescence promotes replication for many viruses, we still lacked evidence on its impact on replication of SARS-CoV-2. Since p21 has been shown to be a key initiator and hallmark of cellular senescence program (28, 29), a *CDKN1A-*knockdown (*CDKN1A^sh^*) cell line was generated to determine whether p21 mediated SARS-CoV-2 induced VIS (**Supplementary Figures 3A, B**). The results showed that SARS-CoV-2 infection failed to induce senescence in *CDKN1A^sh^* cells (**Supplementary Figure 3C**), consistent with the previously reported results observed in *TP53^sh^* cells (14). Furthermore, viral replication in *CDKN1A^sh^* cells was significantly slower than wild-type cells (**Supplementary Figure 3D**). Conversely, overexpression of p16, another initiator of senescence, accelerated the replication of SARS-CoV-2 (**Supplementary Figures 3E, F**), and ribociclib, a small chemical inhibitor of CDK4/6, enhanced viral replication in a dose-dependent manner (**Supplementary Figure 3G**). These findings together confirmed that cellular senescence promoted the replication of SARS-CoV-2.

Therefore, we proposed that SARS-CoV-2 infection triggered a tug-of-war between the host and the virus over cell fate determination: upon infection by SARS-CoV-2, the apoptotic pathway was initiated by host cells as part of the antiviral response, as evidenced by caspase 3 cleavage and activation; however, SARS-CoV-2 blocked apoptosis while driving the cells into a senescent state (**Figure 1**; **Supplementary Figure 2**). The results of this transition created a stable cellular environment conducive to viral replication.

### 3.2 NSP5 mediates cleavage of DAP5 and inhibits apoptosis

We next set out to elucidate the mechanism underlying the inhibition of apoptosis by SARS-CoV-2. Since cleavage of caspase 3, the initiation step of apoptosis, was evident in SARS-CoV-2 infected cells, but the cells did not undergo apoptosis phenotypically, we decided to focus on downstream effectors of cleaved caspase 3. Among the effectors, DAP5 has been shown to be a target for viral replication and survival program (24).

**Figure 2:**
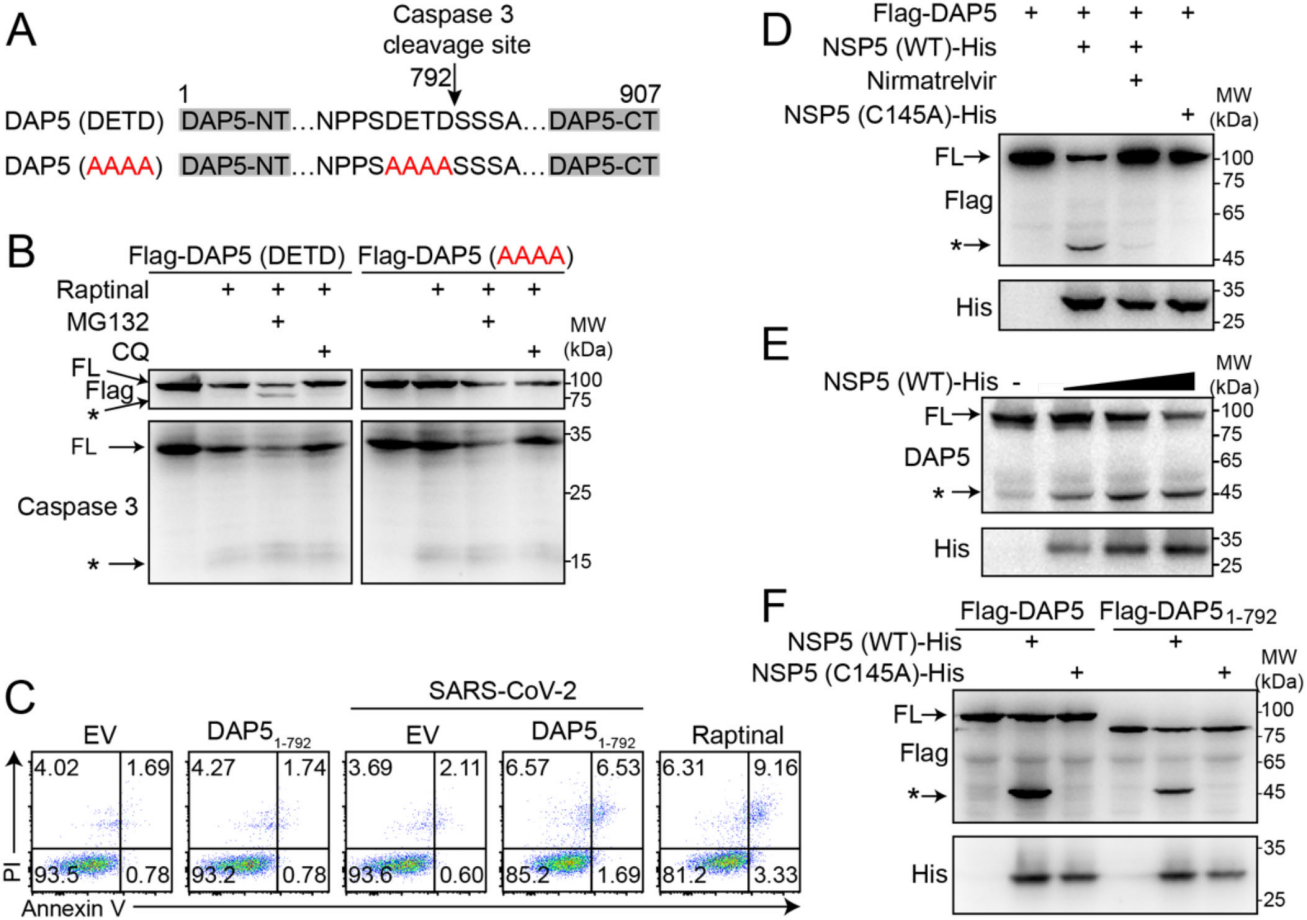
NSP5 cleaves DAP5, distinct from caspase 3 cleavage of DAP5. **(A)** Schematic diagram of the caspase 3 cleavage site (_789_DETD_792_) and mutation (_789_AAAA_792_) in DAP5 protein. **(B)** Western blot analysis of the full-length (FL) and cleaved Flag-DAP5 in HEK293T cells, which were treated with raptinal (3 μM) for 3 hours to induce apoptosis, followed by treatment with the proteasome inhibitor MG132 or autophagy inhibitor CQ for 12 hours. The black asterisk indicates cleaved DAP5 or caspase 3. **(C)** Apoptosis was analyzed by flow cytometry using Annexin V and PI staining in ACE2-HEK293T cells transfected with DAP5_1–792_ overexpression plasmids or the empty vector (EV), with or without SARS-CoV-2 infection (MOI = 0.1) for 48 h. The raptinal-treated group was used as a positive control. **(D)** HEK293T cells were transfected with the indicated plasmids and treated with or without Nirmatrelvir for 24 hours, followed by analysis of DAP5 cleavage by NSP5 or NSP5 (C145A). **(E)** Western blot analysis of the endogenous DAP5 cleavage bands in HEK293T cells overexpressing NSP5 (WT). **(F)** HEK293T cells were transfected with the indicated plasmids, and 24 hours later, cleavage of DAP5 and DAP5_1–792_ by NSP5 (WT) or NSP5 (C145A) was analyzed. Each experiment was independently repeated three times with similar results, and the representative images were shown.

To investigate a possible role of DAP5 here, we constructed a mutant DAP5 with caspase 3 cleavage sequence _789_DETD_792_ was mutated to an _789_AAAA_792_ sequence that is resistant to the cleavage by active caspase 3 (23) (**Figure 2A**). Upon raptinal-induced caspase 3 activation, the cellular level of full-length wild type DAP5-_789_DETD_792_ reduced dramatically with visible cleaved band with molecular weight comparable to that of DAP5_1–792_ when treated by proteasome inhibitor MG132. Whereas, the activated caspase 3 failed to reduce the level of full-length mutant DAP5-_789_AAAA_792_ (**Figure 2B**). Overexpression of DAP5_1–792_ that is the cleaved product of active caspase 3 restored apoptosis upon SARS-CoV-2 infection, demonstrating its pro-apoptotic function (**Figure 2C**).

Inspired by the fact that CVB3 2A protease cleaved DAP5 to enhance viral replication (24), we speculated SARS-CoV-2 might employ a similar mechanism. SARS-CoV-2 encodes two proteases, NSP3 (PLpro) and NSP5 (3CLpro/Mpro). DAP5 has been reported as a potential substrate for NSP5 (30). Sequence analysis revealed four NSP5 cleavage motifs (LQ) within DAP5 (**Supplementary Figure 1**). Upon SARS-CoV-2 infection, cleavage of endogenous DAP5 was observed in cells. This cleavage event was blocked by the NSP5 inhibitor Nirmatrelvir, suggesting that SARS-CoV-2 cleaves DAP5 via its NSP5 protease (**Supplementary Figure 4A**). The interaction of DAP5 with NSP5 at protein level was detected (**Supplementary Figures 4B, C**). Both exogenous and endogenous DAP5 were cleaved by NSP5, generating a ~50-kDa N-terminal fragment (**Figures 2D, E**), which is dramatically different from ~90-kDa DAP5_1-792_. The enzymatic activity of NSP5 was dependent on its catalytic residue C145 (31), and mutation of C145 abolished its protease activity (**Figure 2D**). Further analysis confirmed that NSP5 cleaved DAP5 into ~50-kDa N-terminal and ~52-kDa C-terminal fragments (**Supplementary Figure 4D**), collectively matching molecular weight of full-length DAP5, which is ~102-kD, indicating a single NSP5 cleavage site within DAP5. Additionally, the pro-apoptotic DAP5_1–792_ was also cleaved by NSP5 (**Figure 2F**). These results revealed that NSP5, a SARS-CoV-2 encoded endopeptidase, was capable of cleaving both DAP5 and DAP5_1–792_ at a specific site. The cleavage not only prevented the accumulation of pro-apoptotic DAP5_1–792_ but also processed DAP5/DAP5_1–792_ into distinct ~50-kDa N-terminal fragments, effectively blocking apoptosis pathway.

### 3.3 NSP5 cleaves DAP5 at Q451

**Figure 3:**
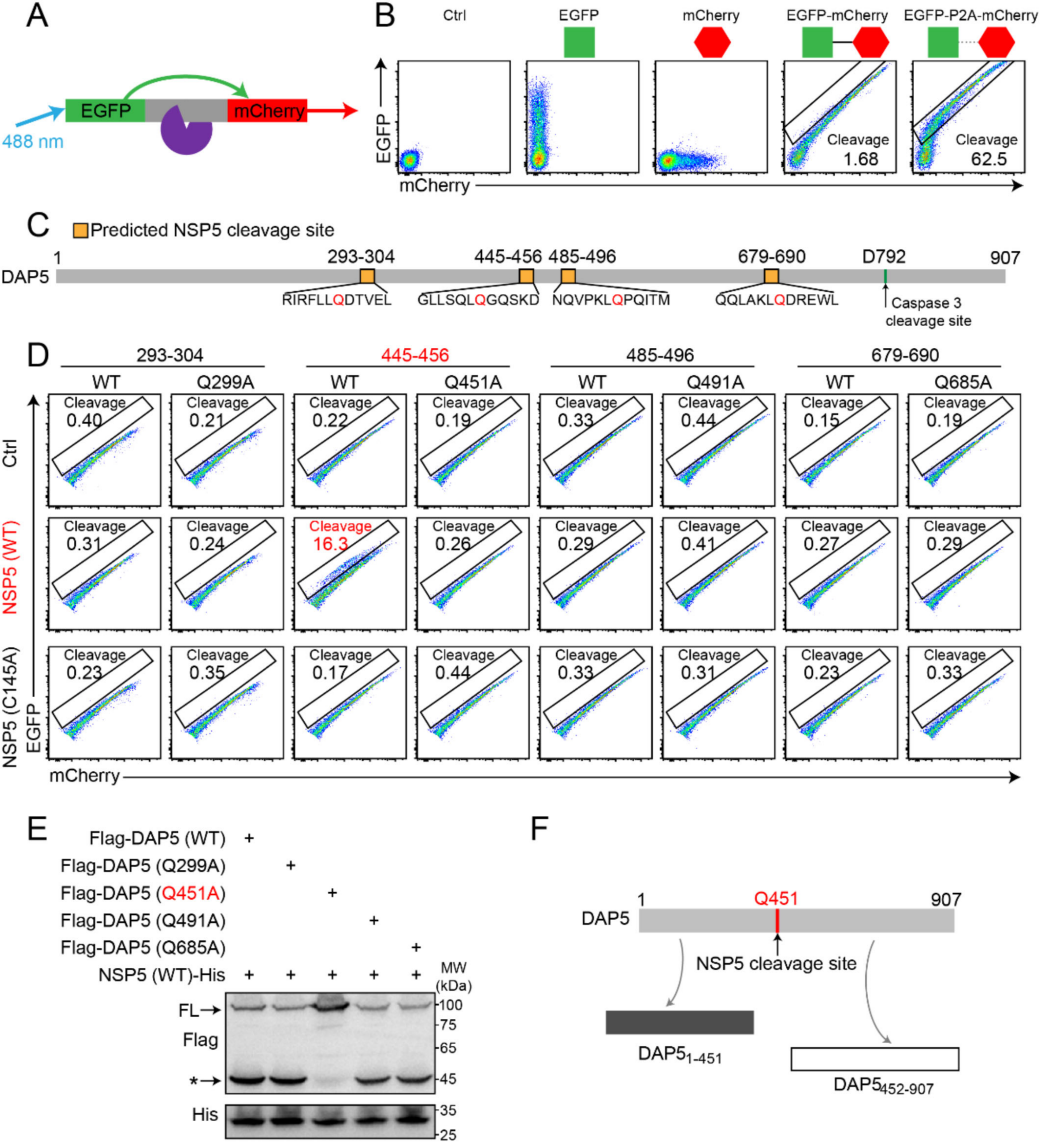
NSP5 cleaves DAP5 at Q451, generating cleavage products DAP5_1–451_ and DAP5_452-907_. **(A)** Schematic diagram of the FRET system using EGFP and mCherry as fluorescent protein pairs. **(B)** Flow cytometry analysis to evaluate the feasibility of the FRET system. **(C)** Prediction of potential NSP5 cleavage sites on the DAP5 protein. **(D)** Representative flow cytometry analysis of potential NSP5 cleavage sites in HEK293T cells transfected with FRET system plasmids and NSP5 (WT) or NSP5 (C145A). **(E)** Cleavage analysis of NSP5 on DAP5 and DAP5-mutants in HEK293T cells. The black asterisk indicates cleaved DAP5. **(F)** Schematic of the NSP5 cleavage sites on DAP5 and the resulting cleavage products. Each experiment was independently repeated three times with similar results, and the representative images were shown.

To identify the NSP5-specific cleavage site among the four potential sites on DAP5/DAP5_1-792_, we developed a FRET-based system with EGFP as the donor and mCherry as the acceptor fluorescence molecules (**Figure 3A**). The feasibility of this system was validated, with the “Cleavage” gate representing the proportion of cleaved fluorescent proteins (**Figure 3B**). Based on the screening for NSP5 recognition and cleavage motif, potential cleavage sites in DAP5 were predicted at Q299, Q451, Q491, and Q685 (**Supplementary Figure 1**). A set of 12-amino-acid peptides flanking each of the four sites was inserted as a linker between EGFP and mCherry on the vector to construct fluorescent fusion proteins to test the cleavage of these sites by NSP5 in the intracellular FRET system (**Figure 3C**).

Among the four potential cleavage sites, Q451 was identified as the one, based on the followings (1): NSP5 recognized and cleaved the DAP5_445–456_ peptide (**Figure 3D**) (2); mutation of Q451 within DAP5_445–456_ abolished NSP5 cleavage (3); point mutation in DAP5-Q451A rendered it resistant to NSP5 cleavage, consistent with FRET results (**Figure 3E**) (4); *in silicon* structure biology analysis by AlphaFold3 showed interaction between NSP5’s catalytic residue C145 and Q451 in DAP5 (**Supplementary Figure 4E**). These data collectively identified Q451 as the cleavage site for NSP5 on DAP5, resulting in two cleaved products: DAP5_1–451_ and DAP5_452-907_ (**Figure 3F**).

### 3.4 NSP5-mediated DAP5 cleavage induces cellular senescence and enhances viral replication

Next, we investigated whether NSP5-mediated cleavage of DAP5 induced cellular senescence. The mutation of Q451 on endogenous DAP5 effectively preventing its cleavage by NSP5 (**Supplementary Figures 5A, B**). In these DAP5-mutant cells, neither SARS-CoV-2 infection nor NSP5 overexpression was able to induce cellular senescence (**Supplementary Figures 5C, D**), suggesting cleavage of DAP5 at Q451 by NSP5 was essential for SARS-CoV-2 induced VIS. Building on the critical role of DAP5 cleavage in VIS, we next assessed its impact on viral replication. SARS-CoV-2 replication was significantly reduced both in *DAP5^sh^* and DAP5-mutant cells. Notably, this defect was rescued by re−expression of wild−type DAP5, but not by the NSP5 cleavage−resistant mutant DAP5 (Q451A) (**Supplementary Figure 5E**). These results collectively demonstrated that NSP5-mediated cleavage of DAP5 was required for its pro−viral function.

**Figure 4:**
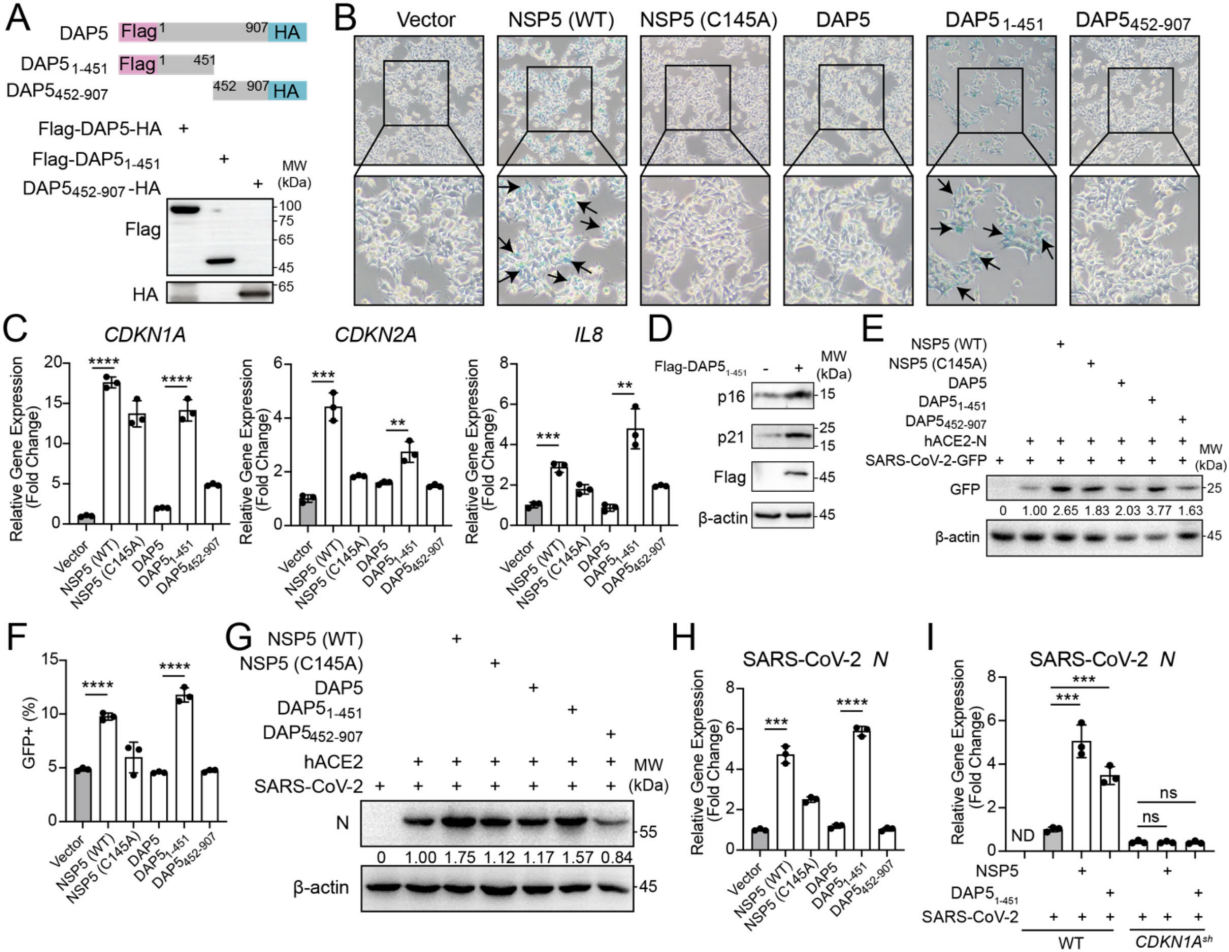
DAP5_1–451_ promotes viral replication by inducing cellular senescence. **(A)** Construction and expression analysis of Flag-DAP5-HA (DAP5-FL), Flag-DAP5_1-451_, and DAP5_452-907_-HA expression plasmids. **(B)** SA-β-gal staining of HEK293T cells transfected with the corresponding plasmids. Senescent cells are stained blue, as indicated by arrows. **(C)** The expression levels of senescence-associated genes in HEK293T cells transfected with the corresponding plasmids. **(D)** Western blot analysis of p16 and p21 protein expression in lysates of HEK293T cells transfected with the empty vector or Flag-DAP5_1–451_ overexpression plasmid. **(E, F)** The GFP protein level **(E)** and the percentage of GFP^+^ cells **(F)** in ACE2-N-HEK293T cells transfected with the indicated plasmids for 12-h, then infected with SARS-CoV-2 GFP/ΔN trVLP (MOI = 1) at 24-h post-infection. **(G, H)** The protein **(G)** and RNA **(H)** expression levels of SARS-CoV-2 Nucleocapsid **(N)** in ACE2-HEK293T cells transfected with the indicated plasmids for 12-h, then infected with SARS-CoV-2 (MOI = 0.1) at 24-h post-infection. **(I)** RT-PCR analysis of *Nucleocapsid* RNA level in cells at 24-h post- SARS-CoV-2 infection (MOI = 0.1) in WT and *CDKN1A^sh^* ACE2-HEK293T cells. Each experiment was independently repeated three times with similar results, and the representative images were shown. Mean ± SEM. **P < 0.01; ***P < 0.001; ****P < 0.0001. ns, not significant. ND, not detected.

To determine which of the two fragments, DAP5_1–451_ or DAP5_452-907_, induced VIS, full-length DAP5 (DAP5-FL), DAP5_1-451_, or DAP5_452–907_ were over-expressed (**Figure 4A**). The results showed that DAP5_1–451_ alone induced senescence-associated characteristics, such as increased β-galactosidase activity and enhanced expression of the senescence-related genes *CDKN1A*, *CDKN2A* and *IL8* (**Figures 4B–D**). The senescence phenotypes were not detected in cells overexpressed DAP5-FL or DAP5_452-907_. Among the three forms of DAP5, DAP5_1–451_ is the only one enhanced viral replication of SARS-CoV-2 GFP/ΔN trVLP (32) and SARS-CoV-2 (**Figures 4E–H**). Furthermore, the pro-viral effect of DAP5_1–451_ was dependent on *CDKN1A* (**Figure 4I**), the key initiator that regulates cell cycle in response to cellular stress and pays central role in the genetic network of cellular senescence. These findings indicated that the NSP5-specific cleavage fragment DAP5_1–451_ induced cellular senescence and facilitated viral replication.

### 3.5 DAP5_1–451_ drives expression of the senescence-driver gene *CDKN1A*

**Figure 5:**
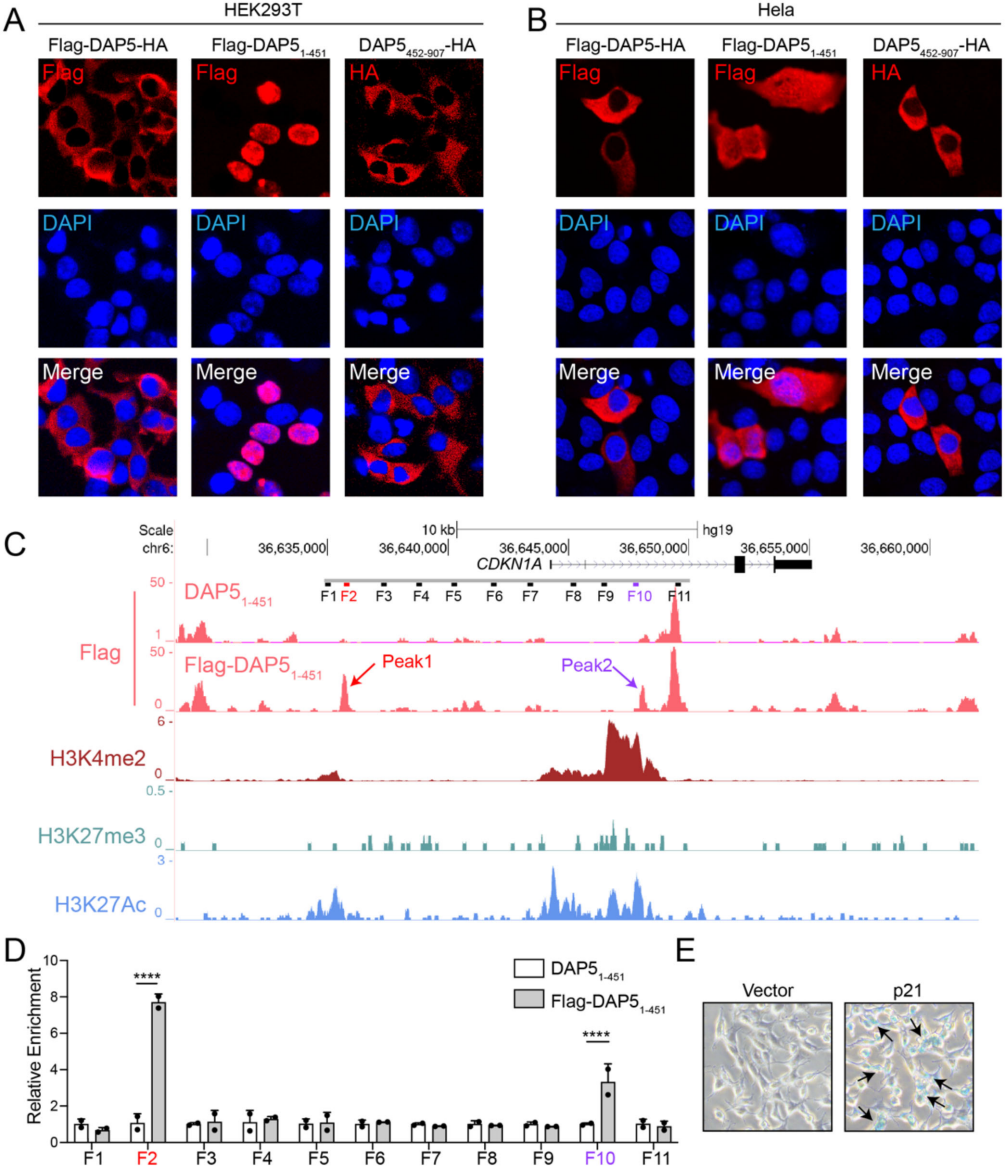
DAP5_1–451_ enters the nucleus and binds near the *CDKN1A* promoter to promote cellular senescence. **(A, B)** Nuclear localization of the indicated DAP5 constructs (Flag-DAP5-HA, Flag-DAP5_1-451_, and DAP5_452-907_-HA) in HEK293T **(A)** and Hela **(B)** cells, as detected by immunofluorescence analysis. **(C)** The binding peaks of Flag-DAP5_1–451_ and the corresponding histone modifications near the *CDKN1A* gene locus. **(D)** ChIP-qPCR validation of DNA regions bound by Flag-DAP5_1-451_. **(E)** The β-galactosidase activity of HEK293T cells with or without overexpression of p21. Senescent cells are stained blue, as indicated by arrows. Each experiment was independently repeated three times with similar results, and the representative images were shown. Mean ± SEM. ****P < 0.0001.

We then set out to investigate the underlying mechanisms by which DAP5_1–451_ induced VIS and facilitated viral replication. Analysis revealed distinct subcellular localization patterns for DAP5 fragments: DAP5_1–451_ primarily localized in the nucleus, while both full-length DAP5 and its C-terminal fragment DAP5_452–907_ were predominantly cytoplasmic (**Figures 5A, B**), which suggested that DAP5_1–451_ might promote cellular senescence as a nuclear protein. Since full-length DAP5 is capable of binding to mRNA in cytoplasm, it was reasonable to speculate that DAP5_1–451_ might bind to DNA in the nucleus. ChIP-seq analysis revealed that DAP5_1–451_ occupancy was enriched at -8 kb on *CDKN1A* that encodes p21 that maintains the genetic program of cellular senescence. We named this enrichment Peak1. Another enrichment of DAP5_1–451_ occupancy at +3 kb, not as intense as Peak1, was named Peak2 (**Figures 5C, D**). Both Peak1 and Peak2 were enriched with histone modifications H3K4me2 and H3K27Ac, markers of *cis*-regulatory elements, especially enhancers (33–35), suggesting the peaks were potential enhancers of *CDKN1A* in response to DAP5_1-451_.

In order to confirm this hypothesis, a dual-luciferase reporter assay was performed (**Supplementary Figures 6A, B**). The reporter plasmids was engineered with Peak1 sequence being inserted upstream of SV40 promoter and Peak2 sequence downstream of luciferase-cDNA, mimicking their physiological positions relative to the *CDKN1A* promoter. Results showed that DAP5_1–451_ enhanced luciferase expression in the presence of Peak1 and/or Peak2, with Peak1 and Peak2 exhibiting synergistic effects (**Supplementary Figure 6C**). Additionally, overexpression of *CDKN1A*-encoded protein p21 induced cellular senescence (**Figure 5E**). In summary, DAP5_1–451_ drove the expression of senescence-driver gene *CDKN1A* by binding to Peak1 and Peak2, two *cis*-regulatory elements on the locus, which resulted in cellular senescence.

### 3.6 DAP5_1–451_ enhanced NF-κB transcriptional activity

Binding of DAP5_1–451_ to the *cis*-regulatory elements near *CDKN1A* promoter to enhance its expression may explain the cell cycle arrest seen in the senescence phenotype induced by SARS-CoV-2. However, this was not an answer to SASP, another hallmark of cellular senescent, which is characterized by the production of pro-inflammatory cytokines, chemokines and matrix metalloproteinases, with NF-κB being the key transcription factor to orchestrate the process (14, 36, 37). Since we (**Figures 1E, G**) and others (14, 16, 19) have shown that SARS-CoV-2 infection induced SASP, we decided to investigate whether DAP5_1–451_ would interact with NF-κB and augment its transactivation activity.

The results showed that SARS-CoV-2 infection resulted in discernible but not pronounced phosphorylation of Ikk, degradation of IκBα, and increased phosphorylation of p65, accompanied by its nuclear translocation (**Supplementary Figures 7A, B**), suggesting a potential activation of the NF-κB signaling pathway upon infection. Furthermore, a luciferase-based NF-κB response element reporter system confirmed that SARS-CoV-2 infection enhanced the transactivation activity of NF-κB (**Supplementary Figure 7C**). DAP5_1–451_ was identified to contribute to NF-κB transcriptional activity based on the following observations (1): expression of NSP5 alone enhanced NF-κB transactivation activity, and co-expression of NSP5 and DAP5 further amplified this effect, indicating that NSP5-mediated cleavage of DAP5 augmented NF-κB activation (**Supplementary Figure 7D**) (2); the cleaved product DAP5_1-451_, rather than full-length DAP5 or the C-terminal fragment DAP5_452-907_, directly enhanced NF-κB transactivation activity, demonstrating that the fragment DAP5_1–451_ represented an active form capable of enhancing NF-κB-dependent gene expression (**Supplementary Figure 7E**) (3); DAP5_1–451_ interacted with p50 (**Supplementary Figures 7F, G**) that forms a heterodimer with p65 to act as a transcription factor in the canonical NF-κB signaling pathway (38). These evidence together suggested the involvement of DAP5_1–451_ in activating the canonical NF-κB pathway.

### 3.7 Nuclear transportation of DAP5_1–451_ dependent on p53

We have found that upon nuclear translocation, DAP5_1–451_ bound Peak1 and Peak2 on *CDKN1A* to drive its expression and enhanced NF-κB transactivation activity to trigger SASP. Although nuclear localization of DAP5_1–451_ was the molecular basis of the results, the mechanism of nuclear import of DAP5_1–451_ remained unclear.

**Figure 6:**
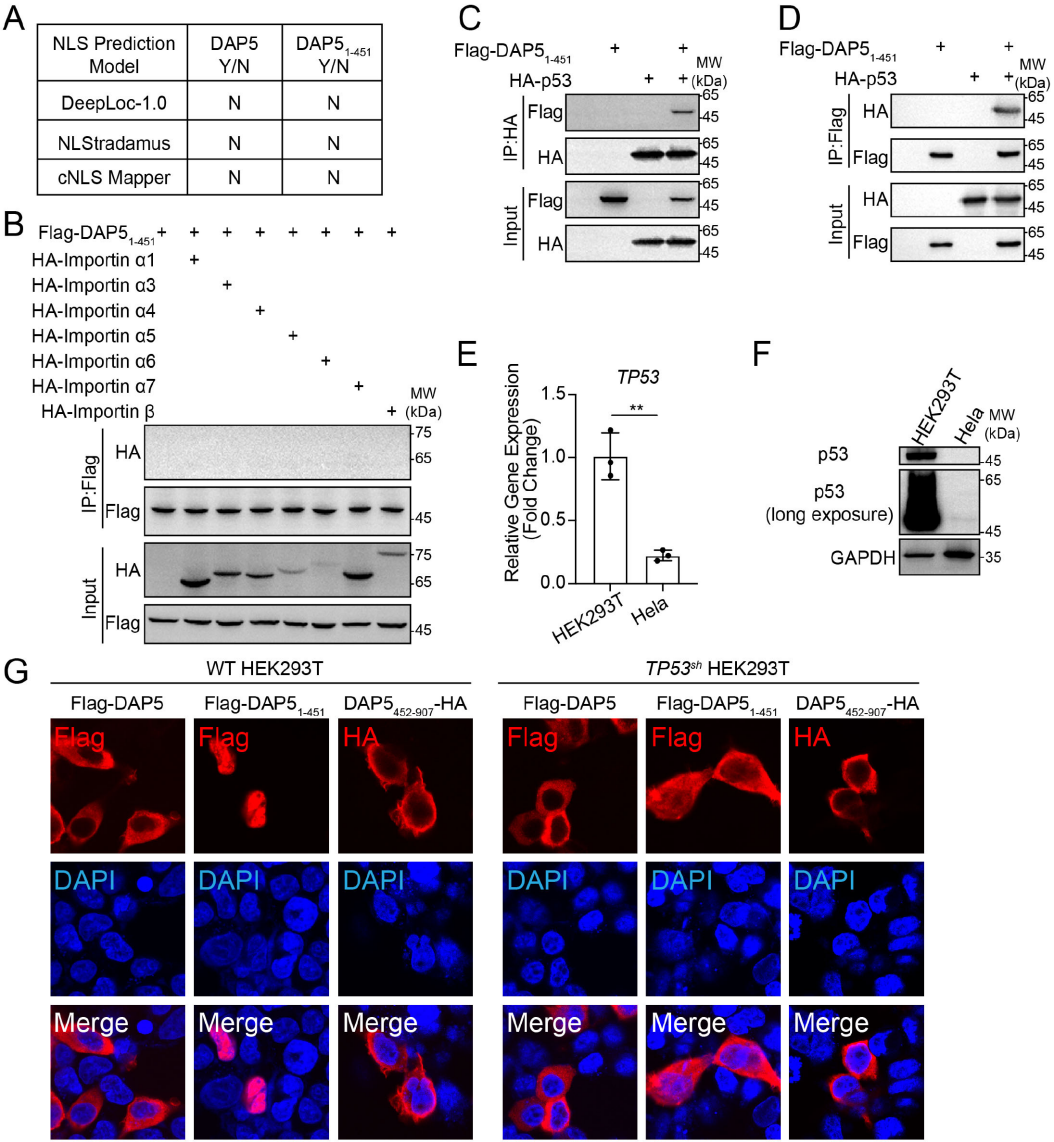
Nuclear localization of DAP5_1–451_ depends on p53. **(A)** Prediction of nuclear localization signals (NLS) in DAP5 and DAP5_1–451_ using DeepLoc-1.0, NLStradamus, and cNLS Mapper. **(B)** Co-IP analysis of the interaction between Flag-DAP5_1–451_ and importin proteins in HEK293T cells. **(C, D)** Assessment of the Flag-DAP5_1–451_ interaction with HA-p53 in HEK293T cells by Co-IP analysis. **(E)** The mRNA level of *TP53* in HEK293T and Hela cells. **(F)** Endogenous p53 expression at the protein level in HEK293T and Hela cells. **(G)** Immunofluorescence analysis of Flag-DAP5, Flag-DAP5_1-451_, and DAP5_452-907_-HA in WT HEK293T and *TP53*-konckdown (*TP53^sh^*) HEK293T cells. Each experiment was independently repeated three times with similar results, and the representative images were shown. Mean ± SEM. **P < 0.01.

Multiple prediction models showed that neither full-length DAP5 nor DAP5_1–451_ contained nuclear localization signal (NLS, **Figure 6A**), nor did DAP5_1–451_ interacted with importin proteins (**Figure 6B**), suggesting the nuclear translocation of DAP5_1–451_ is non-autonomous. We speculated that DAP5_1–451_ migrated to the nucleus through interaction with the transcription factor p53, based on the following facts: (1) DAP5_1–451_ drove cellular senescence by binding to Peak1 and Peak2 on *CDKN1A* locus, which is transcriptionally regulated by p53; (2) Peak1 and Peak2 contained several p53 binding motifs; when the motifs were mutated, the enhancer-like activity of both peaks decreased dramatically in the cells with DAP5_1–451_ over-expression (**Supplementary Figure 6D**); (3) DAP5_1–451_ and p53 interacted with each other (**Figures 6C, D**); (4) in Hela cells whose p53 expression was much lower than HEK293T cells, DAP5_1–451_ showed incomplete nuclear import and remained in the cytoplasm (**Figures 5A, B**, **6E, F**). Furthermore, in *TP53^sh^* HEK293T cells, the nuclear import of DAP5_1–451_ was reduced and it remained in the cytoplasm (**Figure 6G**), suggesting a correlation between p53 levels and DAP5_1–451_ nuclear import, further supporting a p53-dependent import mechanism and explaining the difference between 293T and Hela cells. The results collectively proved that nuclear import of DAP5_1–451_ depended on p53.

### 3.8 TRIM7 enhances antiviral activity by promoting DAP5_1–451_ degradation

**Figure 7:**
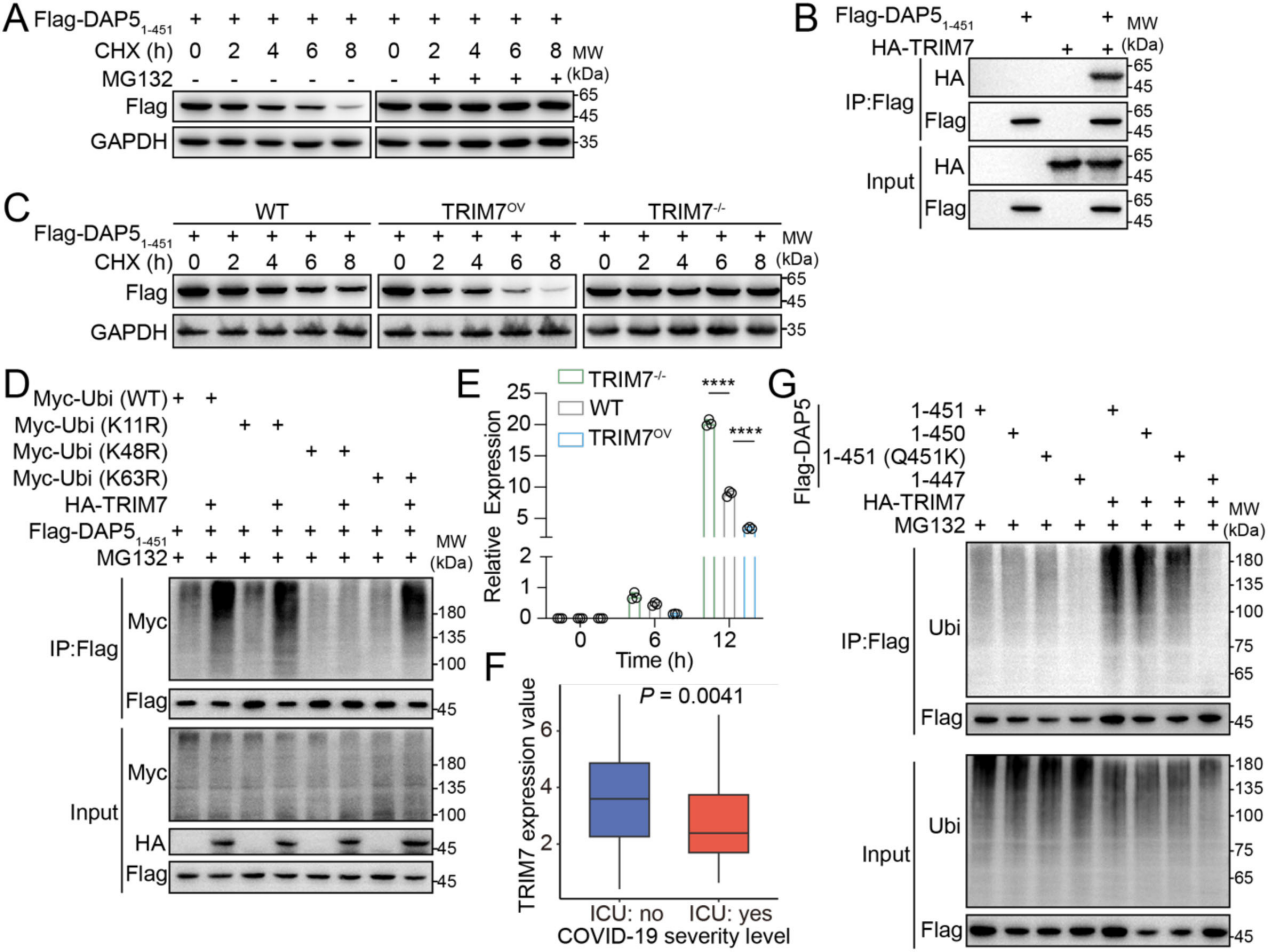
TRIM7 promotes the degradation of DAP5_1–451_ to exert antiviral activity. **(A)** Western blot analysis of Flag-DAP5_1–451_ in HEK293T cells treated with cycloheximide (CHX) and MG132. **(B)** Co-IP analysis of the interaction between Flag-DAP5_1–451_ and HA-TRIM7 in HEK293T cells. **(C)** Cycloheximide chase analysis of Flag-DAP5_1–451_ protein in TRIM7-knockout (TRIM7^-/-^), WT, and TRIM7-overexpression (TRIM7^OV^) HEK293T cells. **(D)** TRIM7^-/-^ HEK293T cells were co-transfected with HA-TRIM7, Myc-Ubi variants (WT, K11R, K48R, and K63R), and the Flag-DAP5_1-451_. The cells were treated with 10 μM MG132 before being collected. Co-IP analysis of the ubiquitin chain linkages on Flag-DAP5_1-451_. **(E)** The RNA level of SARS-CoV-2 *Nucleocapsid* in TRIM7^-/-^, WT, and TRIM7^OV^ ACE2-HEK293T cells at 6 and 12-h post-infection at an MOI of 0.1, as determined by qRT-PCR and calculated relative to *GAPDH*. **(F)** The expression value of the *TRIM7* gene in severe (ICU: yes) and non-severe (ICU: no) COVID-19 patients. **(G)** HEK293T cells were transfected with plasmids encoding Flag-DAP5_1–451_ or indicated mutants in the presence or absence of TRIM7. The ubiquitin conjugation levels were assessed by Co-IP analysis under MG132 treatment. Each experiment was independently repeated three times with similar results, and the representative images were shown. ****P < 0.0001.

SARS-CoV-2 diverted host cell’s fate from apoptosis to senescence *via* DAP5_1–451_ that was a proteolytic product of NSP5-mediated cleavage of DAP5 to promote viral replication. The C-terminal residue of DAP5_1–451_ is Gln, making it a potential substrate for E3 ubiquitin ligase TRIM7 that catalyzes ubiquitination *via* the Gln C-degron we have identified in a previous study (25). Therefore, we hypothesized that ubiquitination and degradation of DAP5_1–451_ by TRIM7 is part of the host’s counter-mechanism against the advantages SARS-CoV-2 gained from DAP5_1-451_. Therefore, we decided to investigate this possibility. DAP5_1–451_ was indeed degraded through the ubiquitin-proteasome pathway (**Figure 7A**). DAP5_1–451_ interacted with TRIM7 and the interaction increased its degradation (**Figures 7B, C**). Further analysis reinforced these findings by revealing that DAP5_1–451_ was ubiquitinated through K48-linkage of ubiquitin, a modification associated with protein degradation, and TRIM7 enhanced the K48-linked ubiquitination of it (**Figure 7D**). Additionally, levels of TRIM7 inversely correlated with the replication of SARS-CoV-2 (**Figure 7E**) in host cells. An inverse correlation of levels of *TRIM7* and disease severity was observed in samples from COVID-19 patients (**Figure 7F**), as well. These results revealed an anti-SARS-CoV-2 function of TRIM7 and suggested that TRIM7 exerted this function by ubiquitination and degradation of DAP5_1-451_.

Since TRIM7 recognizes its substrates *via* Gln C-degron (25), we set out to test whether degradation of DAP5_1–451_ by TRIM7 fits the model. A protein stability analysis system was established using dTomato as an internal reference and fusing DAP5_1–451_ or its mutants to the C-terminal of EGFP. In this system, EGFP/dTomato ratio is the readout of DAP5_1–451_ stability (**Supplementary Figures 8A, B**). By this system it was confirmed that the C-terminal -SQLQ sequence of DAP5_1–451_ mediated its recognition by TRIM7 (**Supplementary Figure 8C**; **Figure 7G**), whereas mutation of the C-terminal Q451 alone in DAP5_1–451_ did not decrease TRIM7’s recognition or degradation of DAP5_1-451_ (**Supplementary Figure 8C**; **Figure 7G**). This finding was a complement to the Gln C-degron of TRIM7 proposed by us (25), because it extended the Gln C-degron from the very last C-terminal residue to the last four residues.

## 4 Discussion

The cellular outcomes of SARS-CoV-2 infection are highly context-dependent, being strongly influenced by cell type, MOI, and infection duration (14, 39–44). Notably, even within the same cell type, distinct cellular fates have been reported depending on the MOI. For example, in Huh7 cells, apoptosis was observed at a higher MOI (MOI = 2), whereas a senescence-associated phenotype was reported at a lower MOI (MOI = 0.1), indicating a dose dependence (42, 44). Consistent with this framework, previous study reported necroptosis in SARS-CoV-2 infected ACE2-A549 cells at an MOI of 0.5, with LDH release becoming evident from 48 hpi and increasing thereafter (39). In the present study, infection of ACE2-A549 cells at a lower MOI (MOI = 0.1) and analysis at early time points (within 48 hpi) revealed a cellular senescence phenotype (**Figures 1D–G**). These observations are considered complementary rather than contradictory, reflecting different stages and intensities of viral infection. In addition, low MOI infection is commonly used to model the initial phase of viral infection, during which only a limited number of virions successfully infect target cells. This approach mimics the early infection stage of respiratory viruses and aligns with the natural infection dynamics described for viruses such as SARS−CoV and influenza virus (45, 46).

Previous studies have reported that SARS-CoV-2 as well as many other viruses could induce VIS, characterized by cell cycle arrest and SASP. However, the underlying molecular mechanisms by which the viruses induce host cell senescence remain unclear. In this study, we systematically investigated this question and revealed that SARS-CoV-2 reprograms fate of host cells through its main protease NSP5, which cleaves the host protein DAP5, thereby blocking apoptosis and inducing VIS. Furthermore, we demonstrated that the host limits this process through a protein degradation system as an antiviral strategy.

The canonical function of DAP5 is an initiation factor involved in cap-independent translation (20). Our findings expand this view by uncovering a stress-sensing and fate-determining role for DAP5 during viral infection. We propose that DAP5 acts as a cellular “homeostasis sensor” that decodes protease signals from either host or viral origin to trigger distinct cell fate programs: cleavage of DAP5 by host proteases such as caspase 3 promotes apoptosis, which facilitates viral clearance; whereas aberrant cleavage by viral proteases such as CVB3 2A protease or SARS-CoV-2 NSP5 promotes viral survival or replication. This “protease signal decoding/integration” mechanism reflects a co-evolutionary arms race between viruses and hosts in controlling cell fate and provides a new conceptual framework for understanding how viruses hijack host homeostatic networks.

We identified that SARS-CoV-2 NSP5 specifically recognizes an LQ motif in DAP5 and cleaves at the Q451 residue (**Figure 3**), consistent with previous reports on substrate preference of NSP5 (47, 48). This is a newly identified cleavage site that differs from those previously reported for caspase 3 or CVB3 2A protease, suggesting that proteases exhibit specificity in recognizing and cleaving DAP5. This feature further supports the functional model of DAP5 as an intracellular “homeostasis sensor” capable of decoding protease signals from either host or virus to determine cellular outcomes such as survival, apoptosis, or senescence. Importantly, the cleavage pattern of SARS-CoV-2 NSP5 toward DAP5 is consistent with the known substrate preference of *Betacoronaviral* NSP5. Sequence alignment and functional assays demonstrated that both SARS-CoV and MERS-CoV NSP5 can cleave DAP5 (**Supplementary Figures 9A, B**), suggesting that NSP5-mediated DAP5 cleavage may represent a conserved mechanism among *Betacoronaviruses* for reprogramming host cell fate, with potential implications for understanding future emerging infectious diseases. While the DAP5 cleavage is conserved across *Betacoronaviral* NSP5, future studies using live SARS-CoV and MERS-CoV will be needed to establish whether the pro−viral function of DAP5_1−451_ represents a general feature within this virus family.

Mechanistically, NSP5 cleavage of DAP5 blocks caspase 3 mediated cleavage (**Figures 2D–F**), thereby preventing the generation of the pro-apoptotic fragment DAP5_1–792_ and inhibiting apoptosis. Critically, the protease activity of NSP5 is a key driver of *CDKN1A* upregulation, despite the observed induction by the catalytically inactive mutant NSP5 (C145A), which hints at additional, protease-independent functions (**Figure 4C**). Meanwhile, the newly generated N-terminal fragment DAP5_1–451_ gains a distinct function: it drives cell cycle arrest and induces SASP, leading to VIS. The VIS further promotes viral replication, which in turn increases the production of NSP5 and enhances its cleavage of DAP5, thereby establishing a self-reinforcing positive feedback loop. Based on this sustained, auto-amplifying mechanism, we infer that the VIS induced under these conditions is likely to represent an irreversible cellular state. However, the strict experimental definition of senescence irreversibility requires removal of the initiating stressor, followed by demonstration that cell-cycle re-entry does not occur. In the context of viral infection, the virus itself constitutes a persistent stressor that cannot be experimentally removed once infection is established. This represents a fundamental technical constraint, precluding a definitive assessment of irreversibility within the experimental system. Notably, DAP5_1-451_, but not the full-length DAP5 or the C-terminal fragment DAP5_452-907_, triggers senescence, suggesting that the C-terminal region may contain an autoinhibitory domain that modulates the N-terminal activity. Future studies on the interactions between different DAP5 domains are needed in order to understand how DAP5 decodes and integrates protease cleavage signals to regulate cell fate determination. Collectively, these findings define a molecular mechanism by which NSP5-mediated DAP5 cleavage reprograms cell fate, suppresses apoptosis, and promotes senescence to support persistent viral infection.

Mechanistic analyses revealed that DAP5_1–451_ interacts with p53 and translocates into the nucleus to activate *CDKN1A* expression, resulting in cell cycle arrest (**Figures 5**, **6**). In parallel, DAP5_1–451_ enhances NF-κB transcriptional activity, and its interaction with p50, together with the preserved activation of a p50/p65-dependent NF-κB reporter (**Supplementary Figures 7E, G**), suggests that DAP5_1–451_ acts to potentiate canonical NF-κB signaling rather than disrupting p50/p65 heterodimer formation. Given that NF-κB is a central transcriptional regulator of the SASP, this enhanced NF-κB activity is likely to contribute to SASP induction. The formation of SASP not only alters the local microenvironment but may also facilitate viral immune evasion and tissue damage by promoting the release of inflammatory cytokines and the recruitment of immune cells (14). However, given the inherent complexity of viral infection and host cellular states, our current experiments do not allow us to formally demonstrate that the DAP5_1-451_-p50 interaction is strictly required for SASP, nor do they exclude the involvement of additional signaling pathways.

The accumulation of DAP5_1–451_ serves as a pivotal molecular event driving the transition from apoptosis to senescence following viral infection, establishing a stable state favorable for viral persistence. Importantly, we have found that DAP5_1–451_ is not a stable protein and is degraded in a TRIM7-dependent manner (**Figures 7A–C**). TRIM7 selectively targets DAP5_1−451_ by recognizing its Gln C−terminal motif (SQLQ) (**Supplementary Figure 8C**). In contrast, full−length DAP5 and DAP5_1−792_ lack such a Gln terminus and escape TRIM7’s degradation, a specificity fully consistent with TRIM7’s established substrate preference. Our previous work showed that TRIM7 targets specific viral proteins of SARS-CoV-2 for degradation (25). Building on this finding, the antiviral function of TRIM7 is not dependent on DAP5. In this study, we demonstrate for the first time that a virus-induced host protein cleavage product can serve as a TRIM7 substrate, expanding the known substrate spectrum of TRIM7. This finding further suggests that the host protein homeostasis system can dynamically regulate aberrant cleavage products induced by viral infection, thereby constituting an intrinsic antiviral defense mechanism.

In summary, our study establishes a “NSP5-DAP5-p53/NF-κB-TRIM7” regulatory network that elucidates how SARS-CoV-2 manipulates the fate of host cells through proteolytic remodeling of host proteins to promote viral replication, while the host counteracts this process via C-degron-mediated protein degradation. This work not only fills a key gap in understanding the mechanism of SARS-CoV-2-induced senescence but also provides broader insight into the conserved pathogenic strategies of *Betacoronaviruses*. Moreover, it highlights potential therapeutic strategies targeting DAP5 cleavage or enhancing TRIM7-mediated degradation to mitigate virus-associated inflammation and persistent infection.

### 4.1 Limitations of the study

The major limitation of this study lies in translating the molecular mechanisms identified in cell models to complex physiological and pathological contexts. Several key questions remain unresolved. First, the *in vivo* relevance of DAP5_1-451_-induced senescence requires validation. It remains to be determined whether DAP5_1–451_ accumulates in infected animal models or patient tissues and exerts measurable effects on host immunity or tissue pathology. Second, in the context of infection, other viral or host proteins may also contribute to senescence, either synergistically or antagonistically, alongside the NSP5-DAP5-p53/NF-κB-TRIM7 axis. Addressing these questions will be critical for integrating this mechanism into the broader landscape of virus-host interactions.

## Data Availability

The datasets GSE157103, GSM3444907, GSM5903250, and GSM5903247 were downloaded from the GEO database (www.ncbi.nlm.nih.gov/geo/). The raw sequencing data generated in this study have been deposited in the NCBI Sequence Read Archive (SRA) database under the BioProject accession number PRJNA1418836.
