# Supplementary material for: Cellular sensor DAP5 decodes Betacoronaviral NSP5 to drive virus-induced senescence
Source: Front Immunol. 2026 Mar 17;17:1768183. doi: 10.3389/fimmu.2026.1768183 (PMC13036145; doi:10.3389/fimmu.2026.1768183)
Supplement: Supplementary file 1 [file DataSheet1.pdf]

Supplementary Online Information

for

Cellular sensor DAP5 decodes *Betacoronaviral* NSP5 to drive virus-induced senescence

By Yao Lu, *et al.*

The PDF file includes:

- Supplementary Figures 1-9 (Figure S1-9)
- Graphic Abstract
- Supplementary Tables 1-6 (Table S1-6)

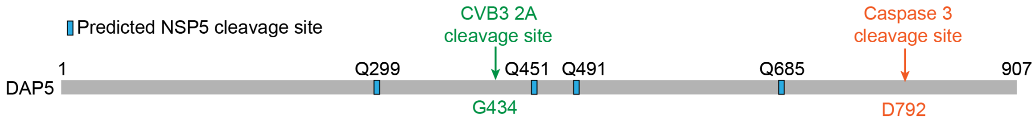

Figure S1. Prediction of potential NSP5 cleavage sites on the DAP5 protein. Bioinformatic analysis of the DAP5 protein sequence for the presence of SARS-CoV-2 NSP5 protease consensus cleavage motifs. The locations of the four queried Gln (Q) residues (Q299, Q451, Q491, and Q685) were indicated. Additionally, the cleavage sites for CVB3 2A protease and Caspase 3 have been annotated on the DAP5 protein.

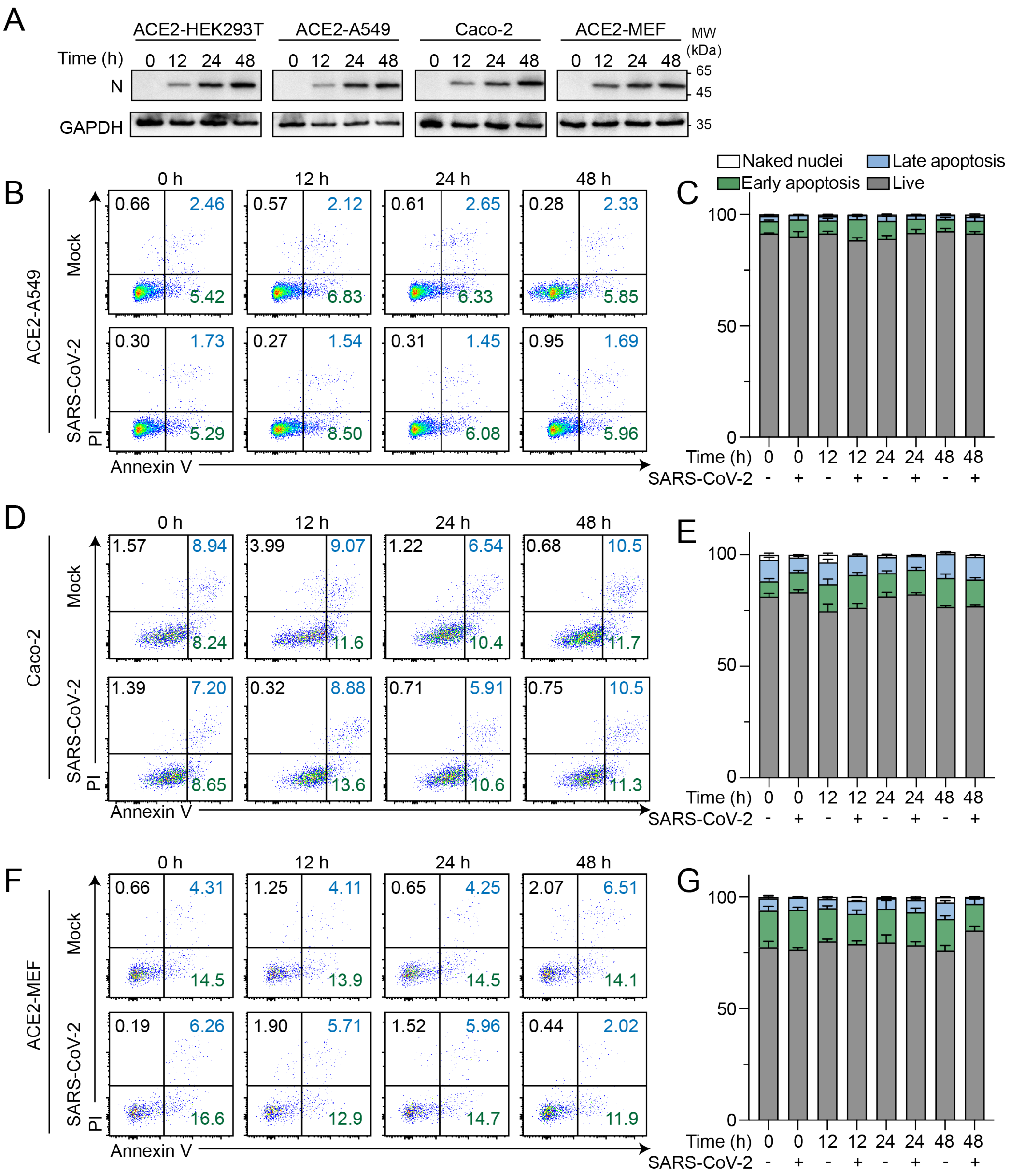

Figure S2. Assessment of apoptosis by Annexin V/PI staining in ACE2-A549, Caco-2 and ACE2-MEF cells following SARS-CoV-2 infection.

(A) Western blot analysis of SARS-CoV-2 Nucleocapsid protein (N) expression in cells infected with SARS-CoV-2 (MOI=0.1) at the indicated time points.

(B-G) Representative flow cytometry and statistical analysis of apoptosis frequency using Annexin V and PI staining in ACE2-A549 **(B, C)**, Caco-2 **(D, E)**, and ACE2-MEF **(F, G)** cells infected with SARS-CoV-2 (MOI=0.1) at 0, 12, 24, and 48 h post-infection.

Each experiment was independently repeated three times with similar results, and the representative images were shown. Mean ± SEM.

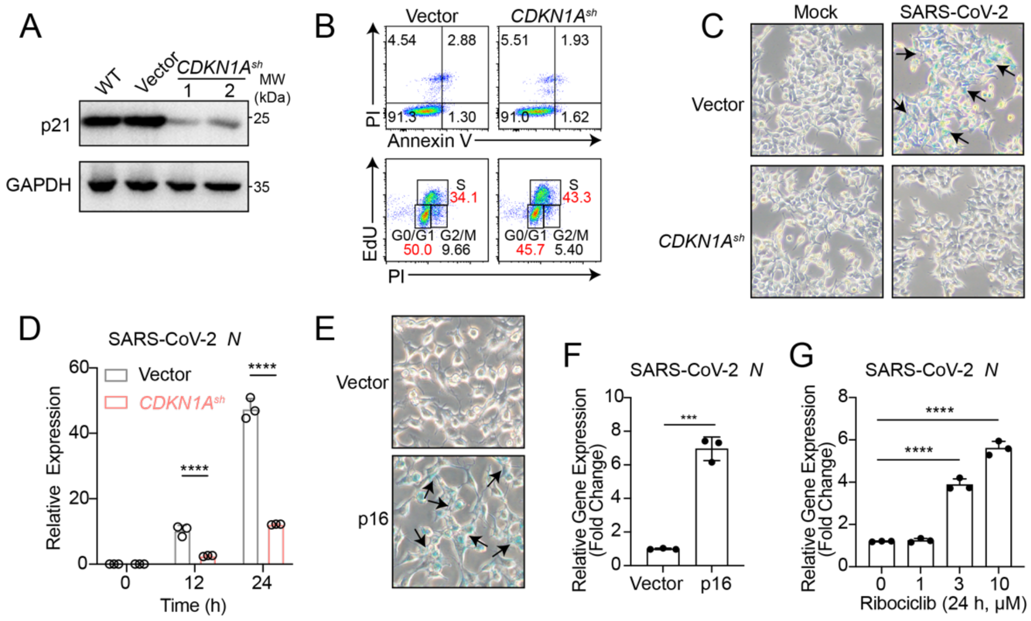

Figure S3. Cellular senescence promotes viral replication.

(A) Western blot analysis of p21 (encoded by *CDKN1A*) in *CDKN1A* knockdown (*CDKN1A*^{sh}) HEK293T cells. The Vector group represents cells transfected with an empty vector as a negative control.

(B) Flow cytometry showing apoptosis assessed by Annexin V/PI staining and cell cycle assessed by EdU/PI staining in Vector and *CDKN1A*^{sh} HEK293T cells.

(C) β-galactosidase activity of Vector ACE2-HEK293T and *CDKN1A*^{sh} ACE2-HEK293T cells 48-hours post-SARS-CoV-2 infection. Senescent cells are stained blue, as indicated by arrows.

(D) SARS-CoV-2 *Nucleocapsid* RNA levels in WT ACE2-HEK293T and *CDKN1A*^{sh} ACE2-HEK293T cells at 0, 12, and 24 hours post-SARS-CoV-2 infection, as determined by qRT-PCR and calculated relative to *GAPDH*.

(E) β-galactosidase activity of ACE2-HEK293T cells transfected with an empty vector or p16 plasmid. Senescent cells are stained blue, as indicated by arrows.

(F) The expression level of SARS-CoV-2 *Nucleocapsid* in ACE2-HEK293T cells with an empty vector or p16 plasmid at 24 hours post-SARS-CoV-2 infection.

(G) The expression level of SARS-CoV-2 *Nucleocapsid* in ACE2-HEK293T cells treated with 0, 1, 3, or 10 μM Ribociclib and then infected with SARS-CoV-2 for 24 hours.

Each experiment was independently repeated three times with similar results, and the representative images were shown. Mean ± SEM. ***$P < 0.001$; ****$P < 0.0001$.

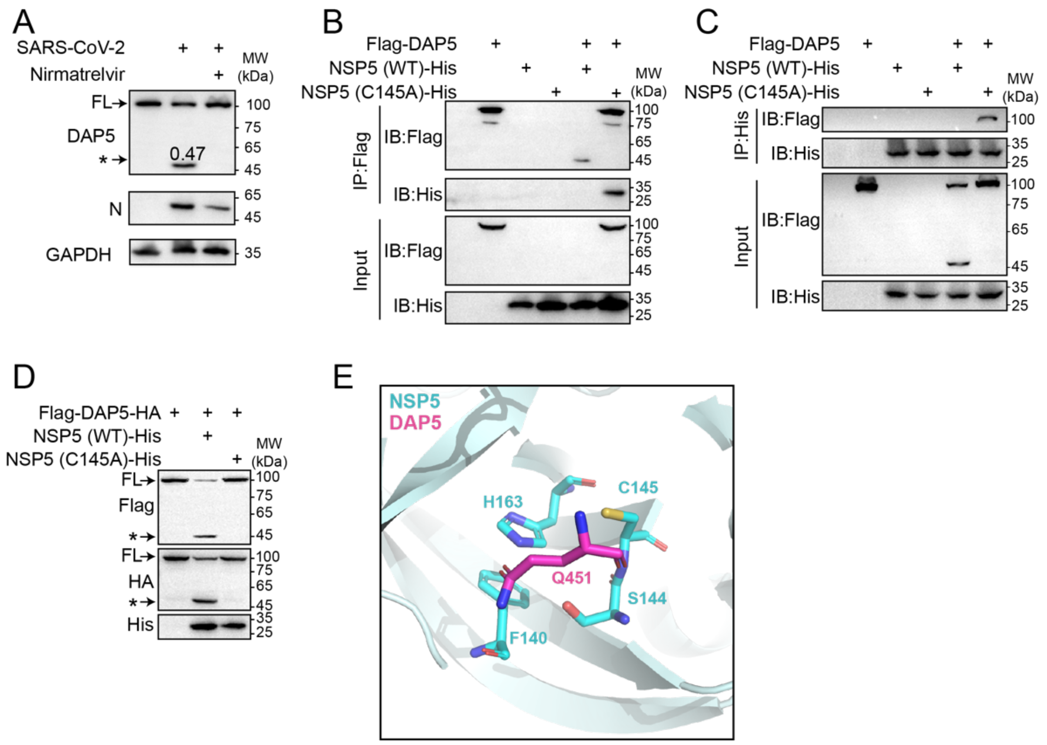

Figure S4. Interaction between NSP5 and DAP5.

(A) Caco-2 cells were pre-treated with or without Nirmatrelvir and then infected with SARS-CoV-2 (MOI=0.1) for 24 h. Cell lysates were analyzed by Western blot for DAP5 cleavage. The cleavage proportion was calculated as: Cleavage proportion = cleaved products / (cleaved products + uncleaved protein).

(B, C) Co-IP analysis of the interaction between Flag-DAP5 and NSP5-His in HEK293T cells.

(D) Western blot analysis of the full-length and cleaved Flag-DAP5-HA in HEK293T cells overexpressing NSP5 (WT) or NSP5 (C145A).

(E) AlphaFold3 prediction of the interaction between DAP5 and NSP5.

Each experiment was independently repeated three times with similar results, and the representative images were shown.

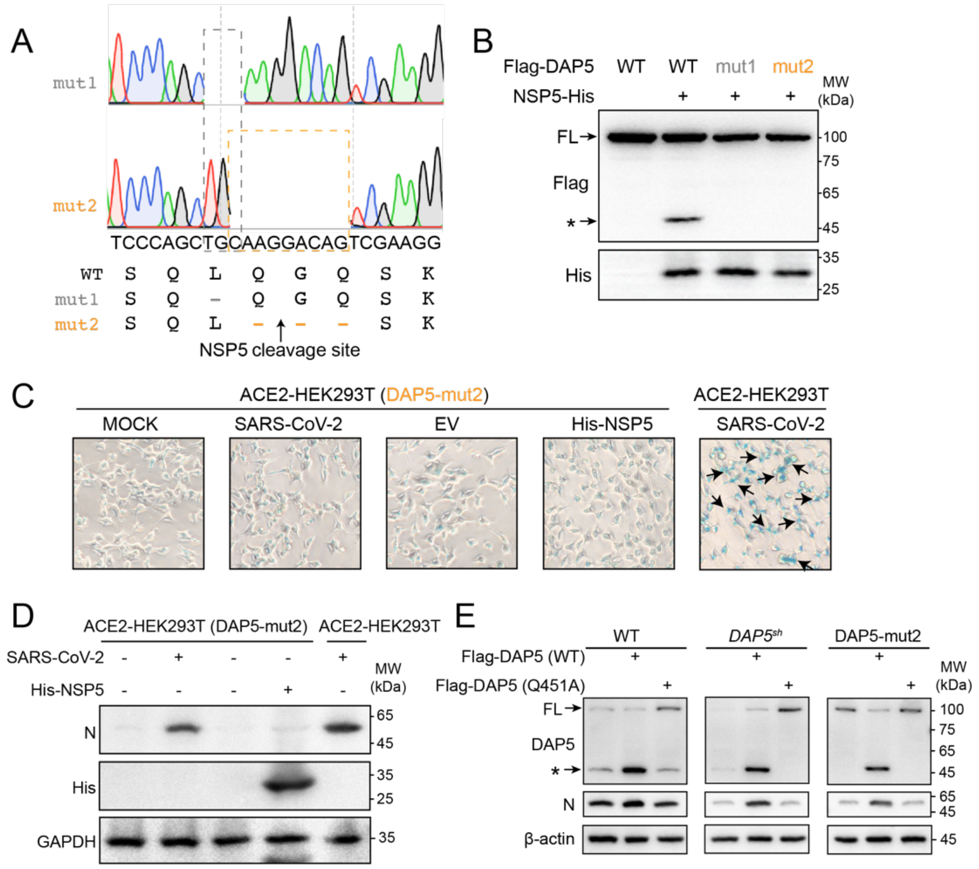

Figure S5. SARS-CoV-2-induced cellular senescence depends on NSP5 cleavage of DAP5.

(A) Sequencing validation of CRISPR-Cas9-mediated mutation of NSP5 cleavage site on the endogenous DAP5.

(B) Western blot analysis of NSP5 cleavage of DAP5 and its mutants. The black asterisk indicates cleaved DAP5.

(C) β-galactosidase activity of ACE2-HEK293T DAP5-mut2 cells. From left to right: uninfected (Mock), SARS-CoV-2 infection, empty vector transfection, and NSP5 overexpression plasmid transfection. SARS-CoV-2 infection in ACE2-HEK293T cells was used as a positive control. Senescent cells are stained blue, as indicated by arrows.

(D) Validation of SARS-CoV-2 infection and plasmid transfection in the experimental samples from **(C)**.

(E) ACE2-HEK293T cells of the indicated genotypes were transfected with plasmids encoding Flag-DAP5 (WT) or Flag-DAP5 (Q451A). 24 h post-transfection, cells were infected with SARS-CoV-2 (MOI=0.1) for 24 h. Cell lysates were analyzed by Western blot. The black asterisk indicates cleaved DAP5.

Each experiment was independently repeated three times with similar results, and the

representative images were shown.

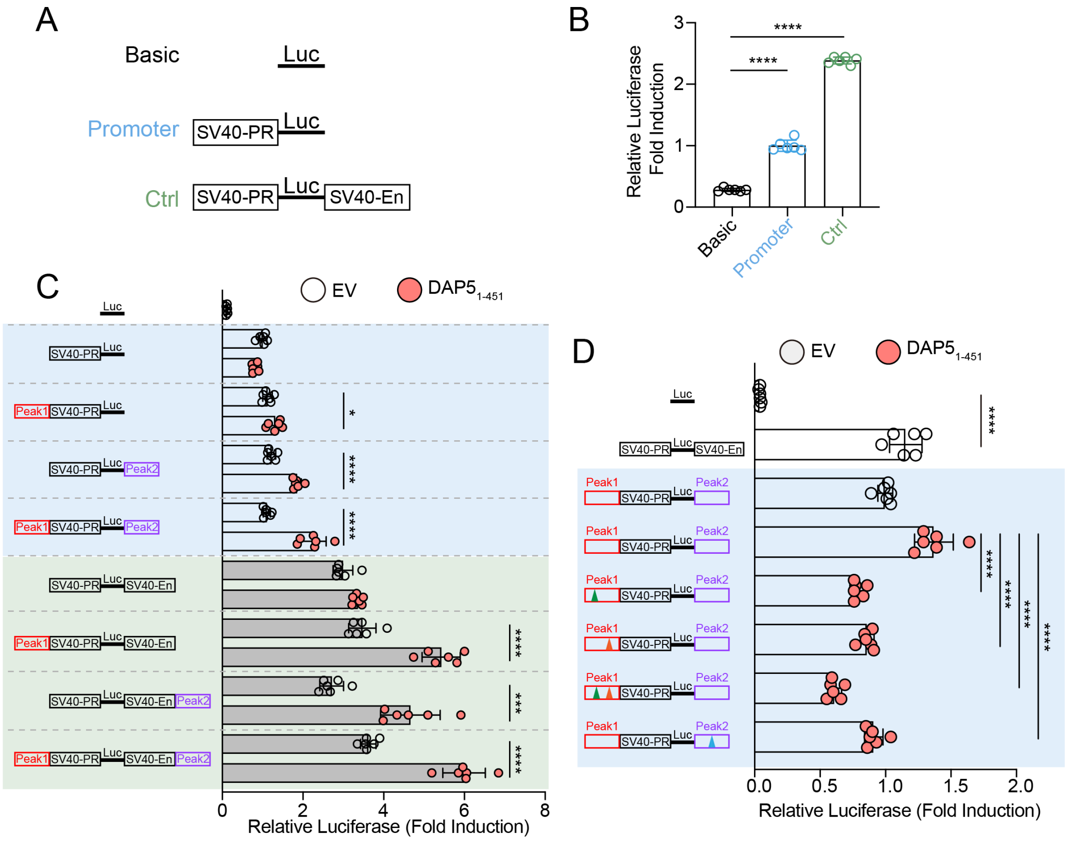

Figure S6. DAP5$_{1\text{-}451}$ binding to the *CDKN1A* promoter depends on the p53 binding site.

(A) Schematic representation of the Basic, Promoter, and Ctrl plasmid elements. Luc represents Firefly luciferase, and the plasmid is pGL3.

(B) Firefly luciferase intensity in HEK293T cells overexpressing the Basic, Promoter, or Ctrl plasmids was measured using a dual-luciferase reporter assay, with Renilla as the internal control.

(C) The Peak1 and Peak2 sequences were inserted into the respective sites of the Promoter or Ctrl plasmids, creating a series of luciferase plasmids. The blue background indicates modifications to the Promoter plasmid, while the green background indicates changes to the Ctrl plasmid. The effect of DAP5$_{1\text{-}451}$ on luciferase expression was assessed using a dual-luciferase reporter assay.

(D) The Peak1-SV40-Luc-Peak2 plasmid was modified by deleting the p53 binding sites in Peak1 or Peak2. The effect of DAP5$_{1\text{-}451}$ on luciferase expression was assessed using a dual-luciferase reporter assay.

Each experiment was independently repeated three times with similar results, and the representative images were shown. Mean ± SEM. *$P < 0.05$; ***$P < 0.001$; ****$P < 0.0001$.

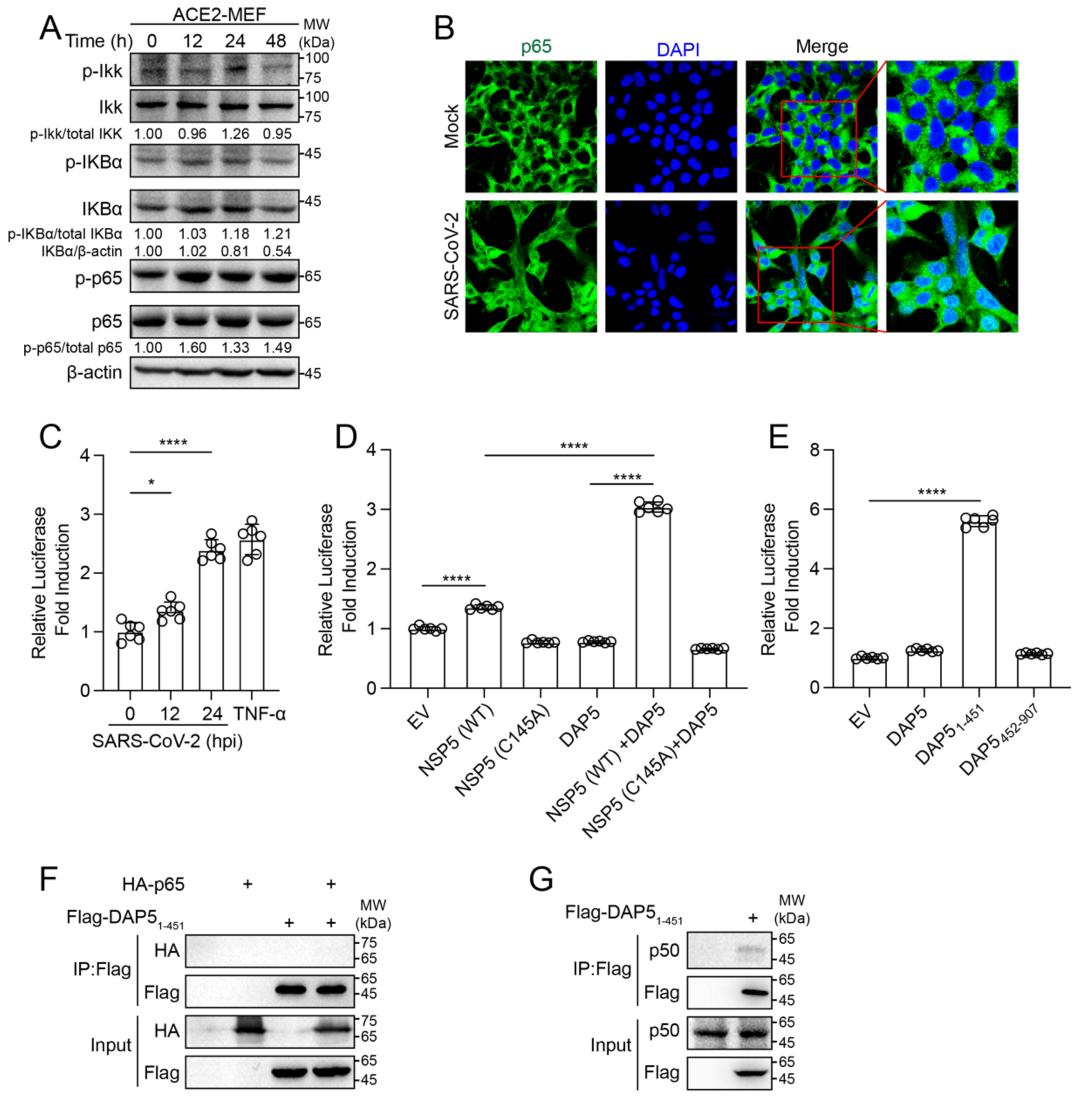

Figure S7. DAP5$_{1\text{-}451}$ enhances NF-κB transcriptional activity.

(A) Western blot analysis of NF-κB pathway activation in ACE2-MEF cells infected with SARS-CoV-2.

(B) Immunofluorescence analysis of p65 expression and localization in ACE2-HEK293T cells infected with SARS-CoV-2.

(C) NF-κB transcriptional activity analysis of ACE2-HEK293T cells transfected with the NF-κB response element-Luciferase-pGL3 plasmid and infected with SARS-CoV-2 for 24 or 48 hours. TNF-α treatment for 12 hours served as a positive control.

(D, E) NF-κB transcriptional activity analysis of HEK293T cells transfected with the indicated plasmids.

(F) Co-IP analysis of the interaction between Flag-DAP5$_{1\text{-}451}$ and HA-p65 in HEK293T cells.

(G) Co-IP analysis of the interaction between Flag-DAP5$_{1\text{-}451}$ and endogenous p50 in

HEK293T cells.

Each experiment was independently repeated three times with similar results, and the representative images were shown. Mean ± SEM. ***P < 0.001; ****P < 0.0001.

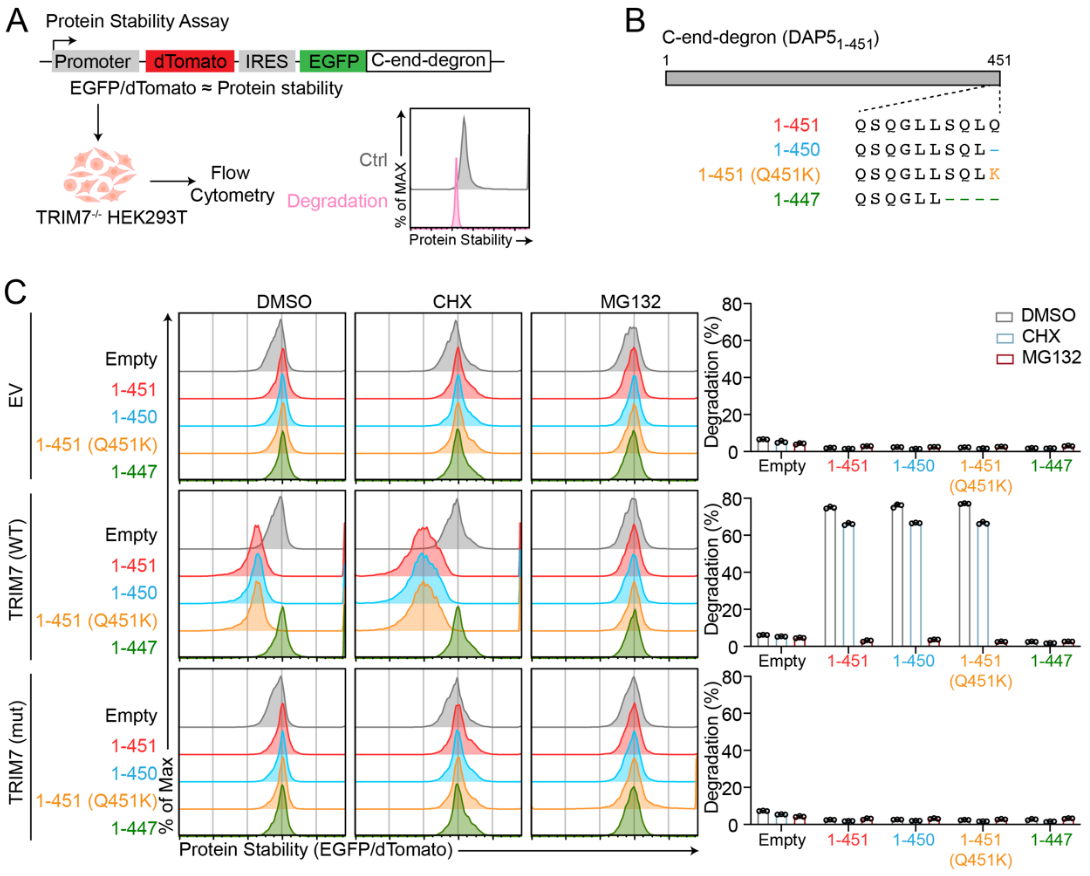

Figure S8. TRIM7 recognizes the C-terminal SQLQ sequence of $DAP5_{1-451}$.

(A) Schematic representation of the Protein Stability Assay system.

(B) Diagram showing the C-degron mutants of $DAP5_{1-451}$, highlighting the last 10 amino acids of the protein sequence.

(C) Flow cytometry analysis of protein stability for $DAP5_{1-451}$ and its mutants in $TRIM7^{-/-}$ HEK293T cells, with quantification of the results.

Each experiment was independently repeated three times with similar results, and the representative images were shown.

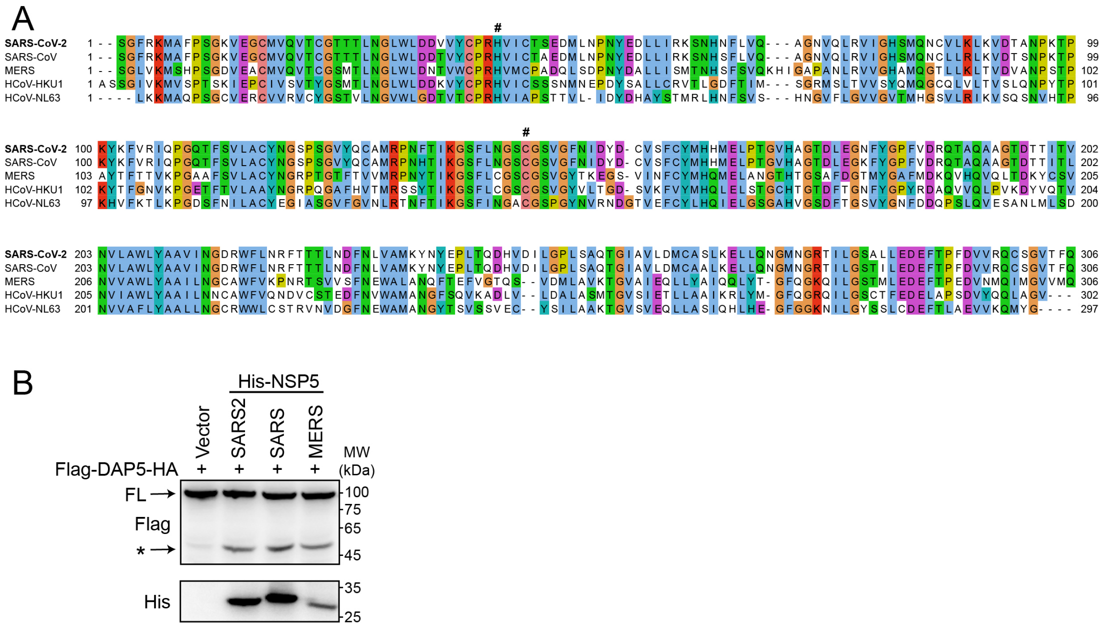

Figure S9. DAP5 cleavage by NSP5 is conserved in coronaviruses.

(A) Sequence alignment of coronavirus NSP5. The # label indicates the enzyme active site of NSP5.

(B) Western blot analysis of the full-length and cleaved Flag-DAP5-HA in HEK293T cells overexpressing coronavirus NSP5. The black asterisk indicates cleaved DAP5.

Graphic Abstract

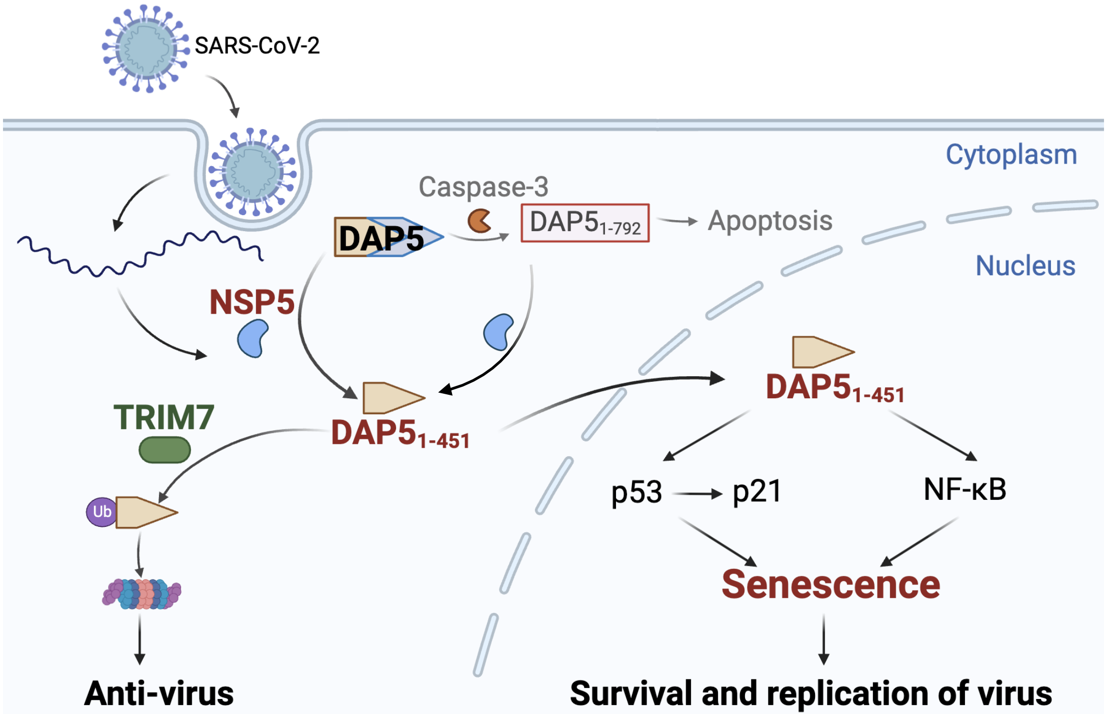

In SARS-CoV-2-infected cells, the apoptotic pathway is activated, leading to the cleavage of DAP5 by Caspase 3 and the production of DAP5$_{1-792}$. The SARS-CoV-2-encoded NSP5 protein inhibits apoptosis by cleaving DAP5 or DAP5$_{1-792}$. The virus-specific cleavage product DAP5$_{1-451}$ induces cellular senescence, thereby promoting viral replication. The host E3 ubiquitin ligase TRIM7 enhances the degradation of DAP5$_{1-451}$ via the ubiquitin-proteasome pathway, exerting antiviral effects. In summary, we describe a cellular-viral "tug-of-war" over the regulation of cell fate centered on DAP5, which involves the cleavage of DAP5 and the degradation of its aberrant cleavage products. This image was created with Biorender.com.

Table S1 List of inhibitors/activators used in cell culture

inhibitor/activator	Source	Catalog
Nirmatrevir	MCE	HY-138687
CQ	MCE	HY-17589A
MG132	MCE	HY-13259
Ribociclib	MCE	HY-15777
Raptinal	MCE	HY-121320

Table S2 List of antibodies used in flow cytometry or WB

Antibodies	Source	Catalog
Caspase 3	Cell Signaling Technology	9662S
SARS-CoV-2 Nucleocapsid	GeneTex	GTX635679
β-actin	Bioss	bs-0061R
p16	Bioss	bs-23797R
p21	Bioss	bsm-60698R
Lamin B1	Bioss	bsm-52361R
p53	Santa Cruz	sc-126
Flag-Tag	Cell Signaling Technology	14793S
His-Tag	Bioss	33004M
HA-Tag	Cell Signaling Technology	3724S
p-Ikkα/β	Cell Signaling Technology	2697
Ikkα	Cell Signaling Technology	11930
p-IKBα	Cell Signaling Technology	2859
IKBα	Cell Signaling Technology	4814
p-p65	Cell Signaling Technology	3033
p65	Cell Signaling Technology	8242
p50	Santa Cruz	sc-8414
GAPDH	Beyotime	AF1186
Myc-Tag	Bioss	bs-23166R
Ubi	Santa Cruz	sc-8017
GFP	Santa Cruz	sc-9996
Zombie NIR Viability Dye	Biolegend	423106

Table S3 List of primers used in RT-PCR experiments

Gene	Sequence
CDKN1A	Forward: 5'- GGGGACAGCAGAGGAAGAC -3'<br>Reverse: 5'- GGCGGATTAGGGCTTCCTC -3'
CDKN2A	Forward: 5'- GGCAGTAACCATGCCCGC -3'<br>Reverse: 5'- TGAAAACTACGAAAGCGGGGTG -3'
IL1A	Forward: 5'- TGGCCAAAGTTCCAGACATG -3'<br>Reverse: 5'- CTACCACCATGCTCTCCTTG -3'
IL8	Forward: 5'- GACAGCAGAGCACACAAGC -3'<br>Reverse: 5'- CACTCCTTGGCAAAACTGC -3'
SARS-CoV-2 *Nucleocapsid*	Forward: 5'- ACCGAAGAGCTACCAGACGA -3'<br>Reverse: 5'- GCCGTCTTTGTTAGCACCAT -3'
Fragment 1	Forward: 5'- GCAAGGCCGCATGATGATG -3'<br>Reverse: 5'- CTCATGGCTGAGTGAGCAGG -3'
Fragment 2	Forward: 5'- CAAGACAGACAGGCTGTGCAC -3'<br>Reverse: 5'- GAAACAGTGGCAATGGCTGC -3'
Fragment 3	Forward: 5'- CTGAGATTCCACACTGCTGTGC -3'<br>Reverse: 5'- AGCTCACAACAGCCTTCTGG -3'
Fragment 4	Forward: 5'- GGGGAGGCAACTACAAAACAG -3'<br>Reverse: 5'- CAGAGTGCCCCACCAACTTC -3'
Fragment 5	Forward: 5'- TCCAGCCTCCATCAGTTCCC -3'<br>Reverse: 5'- CCAGGAAAGGCATCCCTTGG -3'
Fragment 6	Forward: 5'- CAGGACTTCCCCAGCGTG -3'<br>Reverse: 5'- CAGGGATCCACCTCCACG -3'
Fragment 7	Forward: 5'- GGCTGGAGTGCAGGTGG -3'<br>Reverse: 5'- GCAGATCACAGGGTCAGGAG -3'
Fragment 8	Forward: 5'- GCCTGAAAGCAGAGGGGC -3'<br>Reverse: 5'- CAGAAAGGCCACGGCACAG -3'
Fragment 9	Forward: 5'- TGTGTCCAGCGCACCAAC -3'<br>Reverse: 5'- TCAAGGAGGCGGGACCC -3'
Fragment 10	Forward: 5'- GGAAGGGCAAGGGTCTCC -3'<br>Reverse: 5'- GCAGCTGATAGAGGCCCTG -3'
Fragment 11	Forward: 5'- AATGGCACCATCCTGGACTC -3'

	Reverse: 5'- GGAGCTTGCAGTGAGCCG -3'
TP53	Forward: 5'- GGCCCATCCTCACCATCATC -3'
	Reverse: 5'- AGTGCTCGCTTAGTGCTCC -3'

Table S4 Primers used in construction of shRNA plasmids

Primers	Sequence
CDKN1A^{sh} *(sh1)*	F:GATCCGCTCTACATCTTCTGCCTTACTCGAGTAAGGCAGAAGATGTAGAGCGTTTTT<br>R:AATTAAAAACGCTCTACATCTTCTGCCTTACTCGAGTAAGGCAGAAGATGTAGAGCG
CDKN1A^{sh} *(sh2)*	F:GATCGACAGATTTCTACCACTCCAACTCGAGTTGGAGTGGTAGAAATCTGTCTTTTT<br>R:AATTAAAAAGACAGATTTCTACCACTCCAACTCGAGTTGGAGTGGTAGAAATCTGTC
TP53^{sh} *(sh1)*	F:GATCGTCCAGATGAAGCTCCCAGAACTCGAGTTCTGGGAGCTTCATCTGGACTTTTT<br>R:AATTAAAAAGTCCAGATGAAGCTCCCAGAACTCGAGTTCTGGGAGCTTCATCTGGAC
TP53^{sh} *(sh2)*	F:GATCGAGGGATGTTTGGGAGATGTACTCGAGTACATCTCCCAAACATCCCTCTTTTT<br>R:AATTAAAAAGAGGGATGTTTGGGAGATGTACTCGAGTACATCTCCCAAACATCCCTC
DAP5^{sh} *(sh1)*	F:GATCGCCAAAGCCTTAAATTGTGCAACTCGAGTTGCACAATTTAAGGCTTTGGCTTTTT<br>R:AATTAAAAAGCCAAAGCCTTAAATTGTGCAACTCGAGTTGCACAATTTAAGGCTTTGGC
DAP5^{sh} *(sh2)*	F:GATCGCAACACTGAATACTGTAGAACTCGAGTTCTACAGTATTCAGTGTTGCTTTTT<br>R:AATTAAAAAGCAACACTGAATACTGTAGAACTCGAGTTCTACAGTATTCAGTGTTGC

Table S5 sgRNA target sequences of DAP5

sgDAP5	Sequence
sg 1	5'- CTTCGACTGTCCTTGCAGCT -3'
sg 2	5'- GGACTCTTATCCCAGCTGCA -3'
sg 3	5'- CCAGCTGCAAGGACAGTCGA -3'

Table S6 Positions of the Sequences Cloned

Fragments	hg19 Coordinates
Peak1	Chr6:36,635,372-36,636,103
Peak2	Chr6:36,647,364-36,648,765
